# GWAS for Interleukin-1β levels in gingival crevicular fluid identifies IL37 variants in periodontal inflammation

**DOI:** 10.1038/s41467-018-05940-9

**Published:** 2018-09-11

**Authors:** Steven Offenbacher, Yizu Jiao, Steven J. Kim, Julie Marchesan, Kevin L. Moss, Li Jing, Kimon Divaris, Sompop Bencharit, Cary S. Agler, Thiago Morelli, Shaoping Zhang, Lu Sun, William T. Seaman, Dale Cowley, Silvana P. Barros, James D. Beck, Matthias Munz, Arne S. Schaefer, Kari E. North

**Affiliations:** 1 Department of Periodontology, School of Dentistry, University of North Carolina, Chapel Hill, NC USA; 2 Department of Dental Ecology, School of Dentistry, University of North Carolina, Chapel Hill, NC USA; 3 Department of Pediatric Dentistry, School of Dentistry, University of North Carolina, Chapel Hill, NC USA; 4 Department of Epidemiology, Gillings School of Global Public Health, University of North Carolina, Chapel Hill, NC USA; 5 Department of General Practice, School of Dentistry, Virginia Commonwealth University, Richmond, VA USA; 6 Oral and Craniofacial Health Sciences, School of Dentistry, University of North Carolina, Chapel Hill, NC USA; 7 Lineberger Comprehensive Cancer Center, University of North Carolina, Chapel Hill, NC USA; 8 UNC Animal Models Core, University of North Carolina, Chapel Hill, NC USA; 9 Department of Periodontology, Institute of Dental, Oral and Maxillary Medicine, Charité - University Medicine Berlin, Berlin, Germany; 10 Institute for Cardiogenetics, University of Lübeck, 23562 Lübeck, Germany

## Abstract

There is no agnostic GWAS evidence for the genetic control of IL-1β expression in periodontal disease. Here we report a GWAS for “high” gingival crevicular fluid IL-1β expression among 4910 European-American adults and identify association signals in the *IL37* locus. rs3811046 at this locus (*p* = 3.3 × 10^−22^) is associated with severe chronic periodontitis (OR = 1.50; 95% CI = 1.12–2.00), 10-year incident tooth loss (≥3 teeth: RR = 1.33; 95% CI = 1.09–1.62) and aggressive periodontitis (OR = 1.12; 95% CI = 1.01–1.26) in an independent sample of 4927 German/Dutch adults. The minor allele at rs3811046 is associated with increased expression of IL-1β in periodontal tissue. In RAW macrophages, PBMCs and transgenic mice, the *IL37* variant increases expression of IL-1β and IL-6, inducing more severe periodontal disease, while IL-37 protein production is impaired and shows reduced cleavage by caspase-1. A second variant in the *IL37* locus (rs2708943, *p* = 4.2 × 10^−7^) associates with attenuated *IL37* mRNA expression. Overall, we demonstrate that *IL37* variants modulate the inflammatory cascade in periodontal disease.

## Abstract (web summary)

IL-1β in gingival crevicular fluid (GCF) is a marker of inflammation in periodontal disease. Here, Offenbacher et al. identify genetic variants in the *IL37* locus associated with GCF-IL-1β and show that the IL-1β-increasing allele at rs3811046 leads to an enhanced inflammatory response in vitro and in vivo.

## Introduction

Periodontal disease is a common, dysbiotic inflammatory condition. The current understanding of its pathogenesis is based upon an aberrant inflammatory response to the commensal biofilm that drives tissue destruction, a chronic and relatively stable dysbiosis at the biofilm-gingival interface^1,2^. Approximately half of the US adult population has periodontitis and 15% are affected by a severe form of the disease^3^. If left untreated, it leads to tooth loss. Importantly, the disease confers a substantial systemic inflammatory burden^4^ and is associated with numerous comorbidities of systemic conditions^5^.

Periodontal disease offers an opportune model for the study of mechanisms regulating inflammation. Gingival crevicular fluid (GCF) is an atypical, yet easily obtainable and highly informative biomarker fluid that can be used to measure inflammation of periodontal tissues. It is a serum transudate that has been modified by the local host response to the omnipresent bacterial challenge under the gum line; cytokine levels within the GCF reflect the microbial activation of the host’s immune response. IL-1β has been established as a robust GCF biomarker for a hyper-inflammatory phenotype, as well as severe clinical inflammation, bone loss and periodontal disease progression^6–10^. It is a key pluripotent activator of the innate immune response that acts on a myriad of cell types as a trigger for the expression of an expanding cascade of cytokine mediators inducing inflammatory cell recruitment and activation^11^.

IL-1β expression is strongly genetically controlled. Twin studies demonstrated a heritability estimate of 86% for IL-1β response^12^. Previous studies indicate that the IL-1 gene cluster including *IL-1A*, *IL-1B*, and *IL-1RN* loci underlies IL-1β secretion^13^. IL-37, located within the IL-1 gene cluster on 2q12, has recently been identified as a regulatory cytokine that dampens the immune response in humans^14^. These loci have also been reported to be associated with several inflammatory pathogenetic conditions including Alzheimer’s disease^15^, End-stage Renal Disease (ESRD)^12^, osteoarthritis^16^, coronary artery disease^17^, type I diabetes mellitus^18^, stroke^19^, and periodontal disease^16^. However, no evidence on the genetic control of IL-1β exists from agnostic, genome-wide association studies (GWAS) in the context of periodontal disease.

In our current study, we identify *IL37* as the major locus associated with high GCF IL-1β levels. Our findings demonstrate two *IL37* variants with functional roles in decreased expression of IL-37, leading to up-regulation of IL-1β and IL-6, constituting a hyper-inflammatory trait.

## Results

### Characteristics of study population

The demographic, medical and dental characteristics between high and low IL-1β were compared for the study population (Supplementary Table 1). Subjects with high IL-1β levels were more likely to have severe periodontitis, diabetes, high carotid intima-media wall thickness, higher body mass index and to be heavier smokers.

### GWAS Discovery of loci related to GCF-IL-1β response

**Fig. 1:**
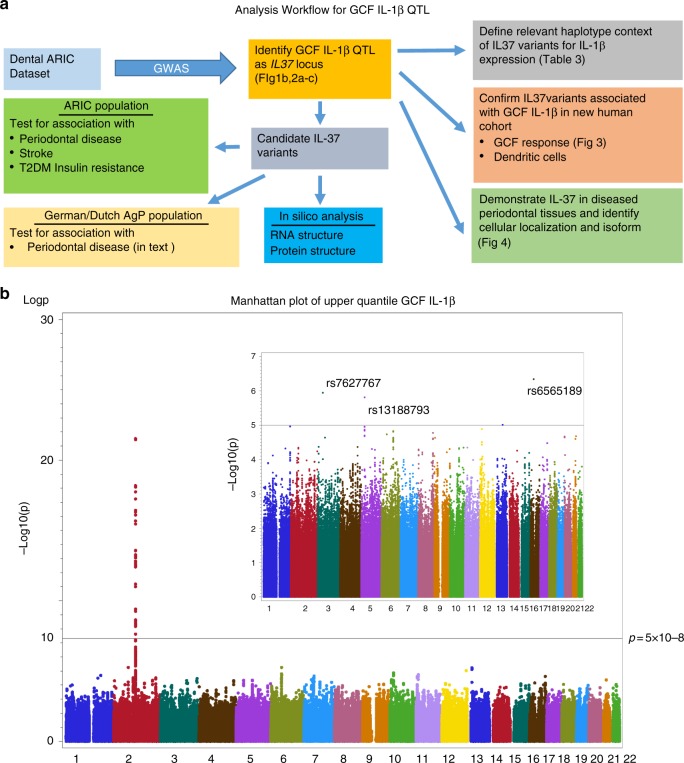
Data analysis workflow and Manhattan plot for GCF and serum IL-1β. **a** Analysis Workflow schema for GCF IL-1β QTL illustrating genomic and phenotype association of GCF-IL1β trait. **b** Manhattan Plot for Top Quartile of GCF IL-1β, as compared to lower 3 quartiles. All 22 chromosomes are shown, plotting the -log10 (*p* values) for each of the 656,292 high-quality SNPS using ProbABEL. For GCF-IL-1β the dominant locus emerges in chr2 with no other genomic regions showing SNPs that cross the significance threshold of *p* = 5 × 10^–8^, as shown as a horizontal reference line. The insert panel represents the Manhattan plot for the serum IL-1β for the same subjects. Although no serum IL-1β SNPs reach GWA significance, the top three are identified in chr 3(rs7627767), 5(rs13188793) and 16 (rs6565189)

The overall analysis workflow for the human studies are summarized (Fig. 1a); which initiates from GWAS analysis of dental ARIC dataset. There was virtually no genomic inflation (Supplementary Fig. 1a and 1b) without significant confounding of the variance of GCF IL-1β level explained by all high-quality genotyped SNPs (*n* = 656,292) after controlling for ancestry, sex, age, and examination center. The entire GWAS results dataset appears as Supplementary Data 1; we have also made it publicly available at http://genomewide.net/public/aric/dental/gcf_il1b/IL1_trait.txt.

After high quality control exclusion, our GWAS found 72 SNPs with *p* < 5 × 10^−8^, highlighting several genome-wide significant loci. Among them, the *IL37* locus within the IL-1 gene cluster is the dominant locus associated with the GCF IL-1β response (Fig. 1b). In contrast, we found no genome-wide significant signals associated with high serum IL-1β levels among the same sample (Fig. 1b, inset).

### The distribution of GCF IL-1β values with discovered loci

Using the ranked value of the mean GCF level of IL-1β, the distribution is plotted for each of the 4907 subjects and indicates a marked inflection beginning near the 75th percentile (Supplementary Fig. 2a). The mean GCF IL-1β levels for the lower 75th percentile show 3-fold magnitude of disparity compared to the upper quartile. This finding prompted us to examine the relationship between our discovered loci and the IL-1β response.

Via the lowest three quartiles of the distribution as a cut point, we created stepwise linear regression models stratified on GCF IL-1β levels and identified two *IL37* variants associated with the highest quartile of the IL-1β response. We also identified two *IL1B* variants for the modest IL-1β increase observed over the lower 3 three quartiles (Supplementary Table 2 and Supplementary Fig. 2 b–d).

The *IL37* SNPs each contribute to higher IL-1β levels within the upper quartile of the IL-1β distribution and have an *r*^2^ of 0.0545, or explain 5.5% of the variance in the IL-1β response (Supplementary Table 2). In contrast, the main locus associated with the IL-1β response among the lower three quartiles of the GCF-IL-1β distribution includes two *IL1B* SNPs variants, an *IL38* and an *IL36G* locus (Supplementary Table 2). This finding suggests that these four loci modulate the IL-1 response with lower levels of IL-1β expression compared to *IL37* locus. We further used the GWA locus zoom plots for the comparisons of IL-1β expression between *IL37* and *IL1B* locus. Our data demonstrated that ‘elevated’ IL-1β (50th –75th percentile versus below median) was mainly driven by variants of the *Il-1B* locus and “high” GCF IL-1β (top *versus* bottom 3 quartiles) was strongly associated with the variants of *IL37* locus (Supplementary Fig. 2 b-d).

### The association of IL-37 Variants with high GCF IL-1β

**Fig. 2:**
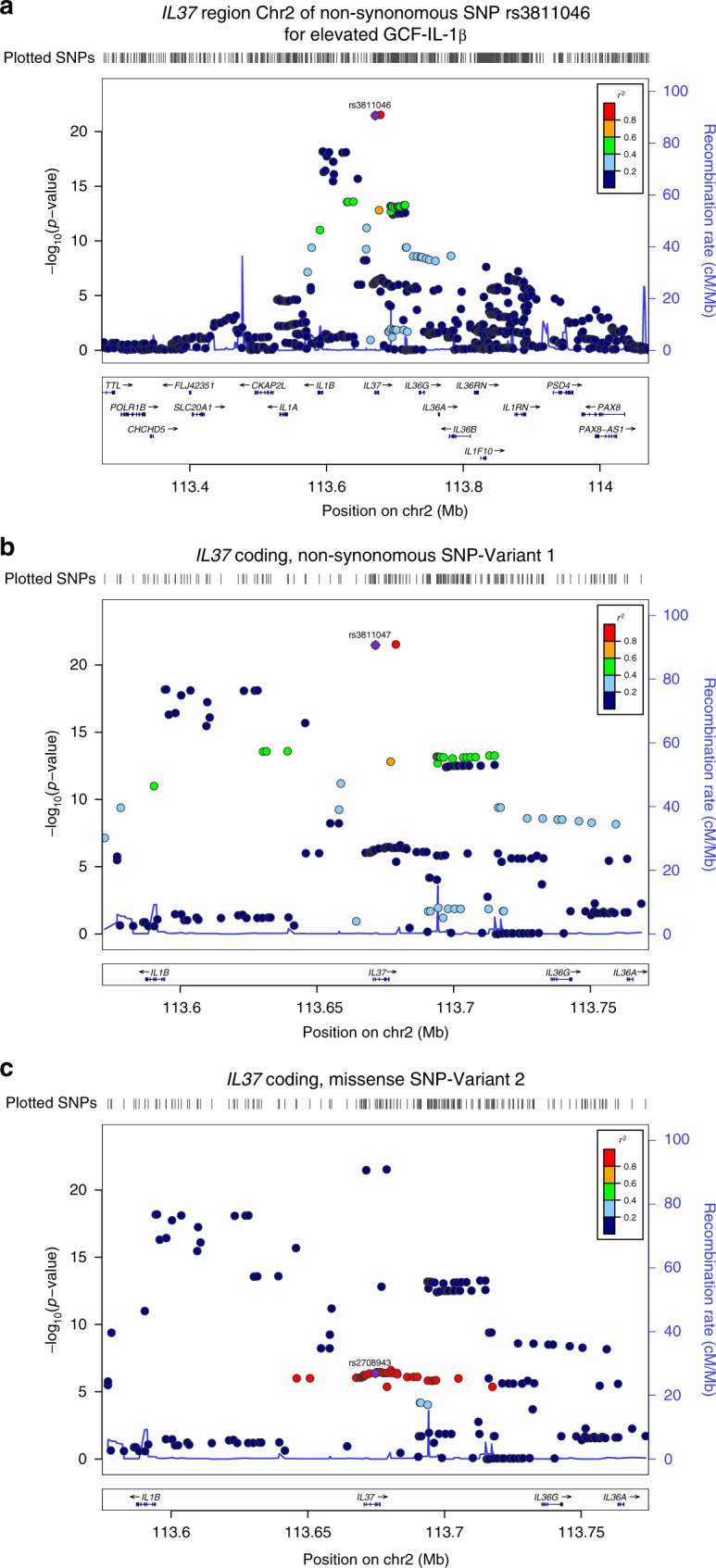
LocusZoom Plot for IL-37 SNPs. The genomic region shown in the Manhattan plot in chr2 is magnified using LocusZoom to illustrate the individual SNPs within the dominant locus for Top Quartile of GCF IL-1β. **a** LocusZoom Plot shows entire IL-1 gene complex region from *IL1A* to *IL1RN*. *IL37* (IL1F7, rs3811046, p = 3.3 × 10^−22^), *IL36G* (IL1F9, rs11677903, *p* = 5.4 × 10^−14^), *IL38* (IL1F10, rs1301182, *p* = 2.4 × 10^−8^), *IL36A* (IL1F6, rs6714534, *p* = 6.7 × 10^−9^) and *IL1B* (rs16944 *p* = 6.5 × 10^−19^). **b** LocusZoom Plot for IL-37 SNP-Variant 1 highlighting the lead SNP at rs3811047 (missense). **c** LocusZoom Plot for IL-37 SNP-Variant 2 that shows additional missense SNPS in disequilibrium with rs2708943 as the indexed SNP

The significant loci in the *IL1* gene complex area of chromosome 2 included *IL37*, *IL36G*, *IL38*, *IL36A* and *IL1B* (Fig. 2a). *IL37* emerged as the top locus for high GCF IL-1β (lead missense SNP: rs3811046, minor allele (G) frequency (MAF) = 0.30, *p* = 3.3 × 10^−22^). This SNP is high LD (D′ = 1.0, *r*^2^ = 0.98) with rs3811047 and both non-synonymous. The coding polymorphisms are within exon 2 and result in a predicted altered IL-37 protein structure of V31G and A42T. In contrast with the major allele at this IL-37 locus, which we term IL-37 Wild Type (IL-37WT), we refer to the variant with both rs3811046 and rs3811047 minor alleles as IL-37 Variant1 (IL-37V1) (Fig. 2b).

Albeit not significant, a second signal within *IL37* (MAF = 0.085) exists corresponding to a haplotype block containing 5 non-synonymous, coding SNPs (rs2708943 (exon 4), rs2723183 (exon 4), rs2723187 (exon 5, lead SNP), rs2708947 (exon 6) and rs2723192 (exon 6); OR = 1.52 (1.31–1.84), *p* = 4.2 × 10^–7^). This polymorphism is in weak LD (*r*^2^ = 0.23) with IL-37V1, and is termed IL-37 Variant2 (IL-37V2) (Fig. 2c). IL-37 mRNA has instability elements which modulate the expression of IL-37^20^. We predicted secondary mRNA structures for these IL-37WT and variants by http://rna.urmc.rochester.edu/RNAstructureWeb/Servers/Predict1/Predict1.html (Supplementary Fig. 3). The IL-37WT and V1 have similar predicted secondary mRNA structures, whereas IL-37V2 and V1V2 have altered predicted secondary mRNA structures demonstrating potentially differing accessibility to the instability sequence of the mRNA structure^20^. Thus, we hypothesized that these polymorphisms may lead to alterations of IL-37 structure, transcription or activation that could result in loss-of-function.

### GCF IL-1β levels among different IL-37 variants

Haplotypes of these two *IL37* variants show the association with GCF-IL-1β levels (Table 1). The presence of the IL-37V1 minor allele is associated with higher GCF-IL-1β levels ((1.1) TT: 134.5 + 3.1 ng/mL, (1.2) TG: 162.4 + 3.5 ng/mL and (2.2) GG: 188.1 + 7.5 ng/mL). The IL-37V2 minor allele does not occur unless IL37V1 has one or more copies of the minor allele (Supplementary Table 2). Interestingly, the effects of V2 in the presence of one or more copies of the minor allele of V1 appears to enhance the effect of the V2 minor allele-increasing the GCF-IL1β level. For example, the GCF-IL1β levels for the dominant variant 1/variant 2 haplotype 1.1/1.1 is 134.5 ng/ml. As compared to 1.1/1.1 for 2.2/1.1, the GCF-IL1β levels increase to 186.8 ng/ml, and for 2.2/2.2 the levels increase further to 253.7 ng/ml. This data suggests that these 2 loci may act synergistically to increase IL-1β.

**Table 1:** GCF- IL-1β levels among IL-37 variants haplotypes

Variant 1^a^ Rs3811046	Variant 2^b^ Rs2723187	*N*	Mean GCF IL-1β ng/ml (StErr)
1.1 (TT)	1.1 (CC)	2181	134.5 (3.12)
1.1 (TT)	1.2 (CT)	0	—
1.1 (TT)	2.2 (TT)	0	—
1.2 (TG)	1.1 (CC)	1304	161.0 (4.03)^*^
1.2 (TG)	1.2 (CT)	515	163.7 (6.42)^*^
1.2 (TG)	2.2 (TT)	0	—
2.2 (GG)	1.1 (CC)	221	186.8 (9.80)^*,***^
2.2 (GG)	1.2 (CT)	155	172.3 (11.70) ^**,****^
2.2 (GG)	2.2 (TT)	32	253.7 (25.75) ^*,*****,#,##^

^a^IL37 Variant 1, lead SNP rs3811046- 1.1 (TT), 1.2 (TG), 2.2 (GG)

^b^IL37 Variant 2 lead SNP rs2723187- 1.1 (CC) 1.2 (CT), 2.2 (TT)

Overall *p* value (GLM) < 0.0001

Pairwise *p* values compared to 1.1/1.1—*p < 0.0001 **p = 0.0018

Pairwise *p* values compared to 1.2/1.1 or 1.2/1.2—***p = 0.01, ****p = 0.004, *****p = 0.0004

Pairwise *p* values compared to 2.2/1.1 ^#^*p* = 0.015, compared to 2.2/1.2 ^##^*p* = 0.004

### IL-37V1 is associated with high GCF innate immune mediators

**Fig. 3:**
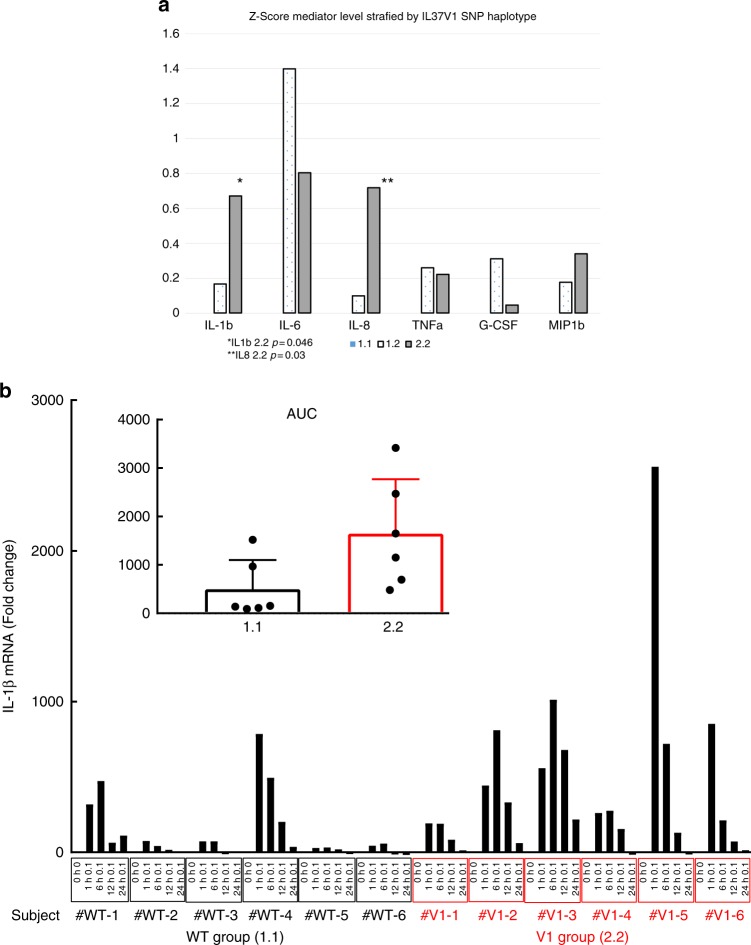
Association of IL-37V1 with GCF Cytokines and DC IL-1β mRNA expression. **a** Z-Score Standardized Log GCF IL-1β Levels by IL-37 Variant 1 Haplotype. This figure reflects the Z-score for each of the six GCF mediators assayed in a sample of 107 subjects who were genotyped for the IL-37 V1 locus by pyrosequencing, assaying six mediators within the same GCF sample for each subject and normalizing to IL-37WT. There was an overall increase in the levels of these mediators expressed as a composite z score for each subject pooling each mediator and transforming using the 1.1 allele as zero (*P* = 0.026) and comparing 1.1 to 1.2 + 2.2. The increased expression of IL-1β and IL-8 were significant by pairwise comparisons comparing 1.1 to 2.2 at *p* = 0.046 and *p* = 0.03. **b** IL-1β secretion at various times by dendritic cells from IL-37WT-1.1 individuals and homozygous IL-37V1-2.2 after LPS stimulation. Levels of IL-1β mRNA (fold expression) are shown at LPS concentration stimulation using isolated peripheral blood monocytes differentiated to DCs from subjects who were pyrosequenced for IL-37WT-1.1 (group 1) or for IL-37V1-2.2 (group 2). The insert shows and overall significant enhanced level of IL-1β mRNA among group 2 (V1 2.2) over time expressed as area under curve (AUC) with standard error bars at *p* < 0.05

IL-37 has broad inhibitory effects on many mediators of the innate immune response including IL-1β^14,21^. To replicate our GWAS findings and investigate whether there was a concordant increase in other mediators of the innate immune response, we explored in a different cohort with 143 subjects to assess the association between IL-37V1 and high levels of IL-1β. We determined *V1* genotypes for 107 individuals by pyrosequencing and measured GCF levels of IL-1β, IL-6, IL-8, TNFα, G-CSF, and MIP-1β by multiplex immunoassay, computing z-scores for each mediator normalizing to the IL-37WT indicated by 1.1 (Fig. 3a). The IL-37V2 minor allele was not analyzed in this context, as the numbers were insufficient in this small group to enable comparisons of inflammatory response in the presence of one or more copies of the IL-37V2 allele (Table 1). Overall, there is a significant increase in these mediators comparing subjects with 1.1 genotype to those with 1.2 + 2.2. Levels of IL-1β and IL-8 are statistically significantly elevated among the IL-37V1 2.2 as compared to IL-37WT 1.1 (Fig. 3a). Thus, there might a significant increase in the broader innate immune response within the GCF for IL-37V1 subjects.

Previous studies have reported that dendritic cells could produce IL-37^22^ and also is target cells of IL-37^23^. Thus, the effects of the IL-37V1 allele as a key regulator of IL-1β are further investigated using human dendritic cells from homozygous IL-37V1, as compared to IL-37WT individuals. Monocytes were isolated from genotyped IL-37 variants, differentiated into dendritic cells and challenged with *E.coli* LPS to measure IL-1β mRNA (Fig. 3b). The results indicate that there is a significant increase in the total IL-1β for those IL-37V1 homozygous subjects compared to the IL-37WT genotype (Fig. 3b).

### Association of IL-37V1 with severe forms of Periodontitis

The IL-37 variants are found to not only be associated with a high inflammatory response, but also associated with more severe clinical manifestations of periodontal diseases. Using the Periodontal Profile Class (PPC) that creates 7-levels of disease^24^, IL-37V1 including both 1.2 and 2.2 is significantly associated with Severe Periodontitis (PPC-G), but this variant is not associated with less severe definitions of disease (Table 2).

**Table 2:** Association of rs3811046 with chronic and aggressive periodontitis

Periodontitis diagnosis	*N*	Odds ratio (95% CI)
Dental ARIC PPC-A (Health)	*n* = 1461	Referent
Dental ARIC PPC-B (Mild disease)	*n* = 811	1.10 (0.92–1.32)
Dental ARIC PPC-C (High Gingival Index)	*n* = 158	1.20 (0.86–1.68)
Dental ARIC PPC-D (Tooth Loss)	*n* = 530	1.10 (0.89–1.35)
Dental ARIC PPC-E (Posterior Disease)	*n* = 827	1.01 (0.84–1.21)
Dental ARIC PPC-F (Severe tooth Loss)	*n* = 474	1.14 (0.91–1.42)
Dental ARIC PPC-G (Severe Disease)	*n* = 237	1.50 (1.12–2.00)
German/Dutch -Healthy controls	*n* = 4210	Referent
German/Dutch -Aggressive Periodontitis	*n* = 717	1.12 (1.01–1.26)

Adjusted for Examination Center, Gender, Age, Diabetes, Smoking (5-levels) and 10 Ancestry PC’s

Rs3811046 genotype: 1.1 (TT) vs 1.2 (TG + 2.2 GG)

Chronic periodontitis: in the Dental ARIC cohort

Aggressive periodontitis: in the German/Dutch sample

We also replicate the findings of *IL37* variants associated with more severe aggressive periodontitis (AgP) in an independent German/Dutch sample^25–29^. We queried the AgP/control GWAS genotypes for association of the potential risk allele rs3811046 of the IL-37 locus. We found a nominally significant association of rs3811046 with AgP (OR = 1.12; 95% CI = 1.01–1.26), adjusted for sex and smoking, suggesting that the candidate *IL37* risk locus may also have a role in the etiology of AgP (Table 2).

### Association of IL37V1 with incident 10-year tooth Loss

10-year incident tooth loss of 3 or more (3+) teeth are stratified by *IL37* genotype and disease severity using PPC categories (Supplementary Table 3). The trend is consistent across all categories of baseline periodontal diagnosis. The predicted probabilities for the loss of 3+ teeth within a ten-year period are shown for the allelic variants of IL-37V1 (Supplementary Table 4). These findings indicate that the specific polymorphism provides risk information in the context of the PPC. For example, there is a 5% risk for tooth loss among periodontally healthy IL-37WT-1.1 genotype subjects, as compared to the severe chronic periodontal disease subjects who are at 24% for 1.1 subjects. In the presence of the IL-37V1-2.2 genotype, the probability of 3+ tooth loss increases to 34% for PPC-G subjects (Supplementary Table 4). These data strongly suggest that the carriage of the IL-37V1 is associated with greater risk for tooth loss.

### Association of IL37V1 with prevalent stroke

Previous studies have implicated high IL-1β as being associated with the inflammatory pathophysiology of stroke, diabetes and cardiovascular diseases^30,31^. We indicate that High GCF IL-1β is associated with prevalent diabetes and thick carotid intimal-medial wall thickness (IMT) (Supplementary Table 1). The association of high GCF-IL-1β with diabetes and thick IMT prompted us to examine whether the IL-37V1 allele was also associated with prevalent diabetes, carotid IMT and prevalent stroke. We found that IL-37V1 is a significant independent contributor to prevalent stroke even in fully adjusted multivariate models (Supplementary Table 5). As stroke is a multi-factorial, polygenic condition, it is likely that the IL-37V1 polymorphism is one of the many polymorphisms associated with prevalent stroke.

### Characterization of IL-37b in human gingival tissue

**Fig. 4:**
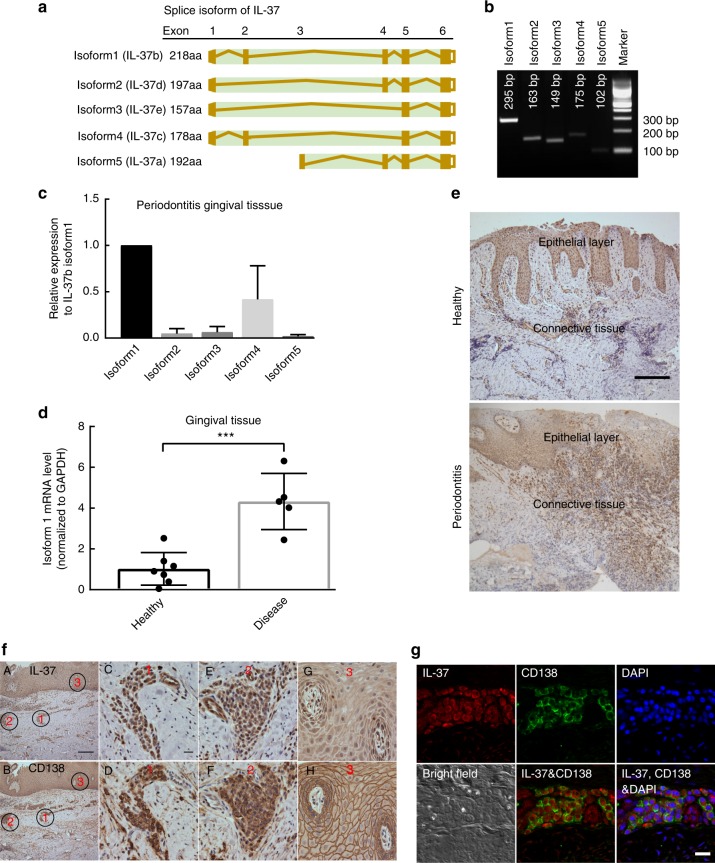
Expression and localization of IL-37 in human gingival tissue. **a** The sketch of five IL-37 isoforms. **b** PCR products amplified with specific primer pairs to different isoforms of IL-37 cDNA. **c** The relative mRNA levels of different isoforms of IL-37 in human gingival tissues from sites with periodontitis were quantitated using real time PCR with isoform-specific primers. **d** The relative mRNA levels of IL-37b (isoform1) in gingival tissues between health and periodontitis (*n* ≥ 5/group). Data show the mean ± SD. ***p < 0.0001 (unpaired two-tailed Student’s *t*-test). **e** The representative level of IL-37 positive staining in epithelial and connective tissue of human gingival tissue from healthy and periodontitis patient using IHC. Scale bar is 200 µm. **f** The localization of IL-37b and CD138 were determined in serial slides of gingival tissue from periodontitis patient. The localization of IL-37 in human gingival tissue was mainly associated with epithelial and infiltrated plasma cells. The black circles with red number indicate representative region of IL-37 positive staining. **A**, **B** represent the low magnification of IL-37 and CD138 staining in human gingival tissue with Scale bar 200 µm. **C**–**F** represent the high magnification of two immune cells infiltrated regions in the connective tissue with Scale bar 20 µm. **g**, **h** represent the high magnification of epithelial cells layer with Scale bar 20 µm. **g** IL-37b and CD138 were co-localized in human gingival tissue of periodontitis patient by immunofluorescence using confocal microscope. The results represent CD138 (green), IL-37 (red), DAPI (blue), and bright field (white). The scale bar is 20 µm. Data shown here are representative of three independent experiments

There are 5 isoforms of IL-37 produced by alternative splicing (Fig. 4a) which have been reported in different tissues^32^. To identify the IL-37 isoforms^32^ existing in human gingival tissue, we designed specific primer pairs for each isoform with amplicons shown in Fig. 4b. The mRNA levels of each different isoform were quantified by real time PCR (qPCR) using inflamed gingival tissue samples from periodontitis patients. Our results indicated IL-37b (isoform1) was the dominant isoform expressed, followed by IL-37c (isoform 4) and the other 3 isoforms were not detected (Fig. 4c). Furthermore, we compare the relative mRNA levels of IL-37b in gingival tissues between healthy and periodontitis subjects, our results indicated that IL-37b levels were significantly higher in periodontitis gingival tissue (Fig. 4d). We additionally determined IL-37 expression and localization by immunohistochemistry (IHC); our results indicated enhanced IL-37 positive staining in periodontitis gingival tissue, especially in the infiltrate within the connective tissue and within the epithelium (Fig. 4e).

We further delineated the major groups of cells producing IL-37 in periodontitis gingival tissue by IHC and found IL-37 mainly expressed by gingival epithelial cells and infiltrated immune cells in connective tissue (Fig. 4f). As the morphology of the infiltrated immune cells in connective tissue displayed a typical plasma cell morphology (eccentric nuclear and voluminous cytoplasm) (Fig. 4f), we investigated the co-localization of IL-37 and plasma cell-specific markers (CD138, stain for both plasma and epithelial cells)^33^. The immunostaining in serial sections using IL-37 and CD138 confirms the infiltrate to be plasma-cell dominant (Fig. 4f). Furthermore, using co-localization of IL-37 and CD138 by immunofluorescence staining following confocal microscopy, we confirmed the major infiltrated immune cells expressing IL-37 are plasma cells, which are known to constitute ~50% of the inflammatory infiltrate in periodontal lesions^34^ (Fig. 4g). In order to confirm this observation we show the colocalization using a second plasma cell-specific confocal microscopy analysis using CD38 (Supplementary Fig. 4a).

To confirm that epithelial cells are the other major source of IL-37 in human gingival tissue, we co-stained IL-37 and an epithelial cell specific marker (Cytokeratin 5) and observed by confocal immunofluorescence microscopy^35^. Our data indicated the colocalization of IL-37 and Cytokeratin 5 (Supplementary Fig. 4b), which indicated that epithelial cells are the other major source of IL-37 in human gingival tissue. To further assess if the DC in human gingival tissue expressed IL-37, we co-stained with IL-37 and DC specific marker (CD11c) and observed by confocal microscopy. Our data show the co-staining of CD11c and IL-37 and indicate that DC in the human gingival tissue are the other source of IL-37 although DC number is not as robust as plasma cells in the human gingival tissues (Supplementary Fig. 4c). Overall, these results from human gingival tissues further suggest the strong association between IL-37 and periodontitis.

### The effect of rhIL-37bWT and Variants on RAW cells and PBMC

**Fig. 5:**
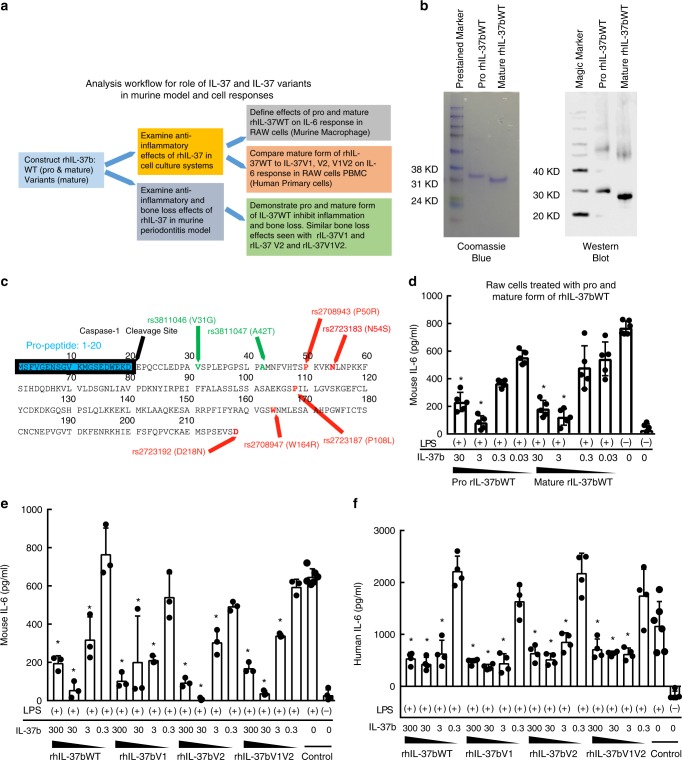
rhIL-37bWT and variants suppress RAW and PBMC response to LPS in vitro. **a** The analysis workflow for role of IL-37 and IL-37 variants in murine and human cell responses and murine periodontitis model. **b** The protein sketch of pro-IL-37b including cleavage site (amino acid 20) and variant sites (green: V1, red: V2). **c** Pro and mature rhIL-37b extracted from *E. coli* were detected on a 12% SDS–PAGE and analyzed by Coomassie blue and western blot using anti-IL-37 antibody. **d** IL-6 production in supernatant of RAW cells treated with gradient concentrations of pro and mature rhIL-37b following LPS (50 ng/ml) stimulation. Data show the mean±SD. *p < 0.05 (One-way ANOVA and Dunnett’s multiple comparisons tests, compare the mean of each group with the mean of LPS alone stimulation group). (+) or (−) indicate the cells with or without LPS stimulation. Results are representative of three independent experiments. **e** IL-6 production in supernatant of RAW cells treated with different concentrations of mature form of WT, V1, V2, and V1V2 rHIL-37b following LPS (50 ng/ml) stimulation. Data show the mean ± SD. *p < 0.05 (One-way ANOVA and Dunnett’s multiple comparisons tests, compare the mean of each group with the mean of LPS alone stimulation group). Results are representative of three independent experiments. **f** IL-6 production in supernatant of human PBMC cells treated with different concentrations of mature form of WT, V1, V2, and V1V2 rHIL-37b following LPS (50 ng/ml) stimulation. Data show the mean ± SD. *p < 0.05 (One-way ANOVA and Dunnett’s multiple comparisons tests, compare the mean of each group with the mean of LPS alone stimulation group). Results are representative of three independent experiments

The experimental workflow of examining the effects of recombinant proIL-37bWT, mature IL-37bWT, as well as mature forms of IL-37V1, IL-37 V2 and IL-37V1V2 in murine RAW cells and Human PBMC responses and the mouse periodontitis model is in Fig. 5a. Previous findings indicated that the primary sequence of IL-37 isoform b shows the full-length protein including the pro-peptide (residues 1–20) and the matured peptide (residues 21–218) (Fig. 5b)^36^. It is important to note the all SNP variants associated with IL37bV1, IL37bV2 and IL37bV1V2 are in the mature IL-37 region based upon caspase-1 cleavage. Previous studies also reported that the mouse does not express IL-37 or any IL-37 homologs, but does exhibit IL-37 receptor^36,37^. To investigate the function of IL-37b in the development of periodontitis, we expressed and purified both pro and mature form of recombinant human IL-37b (rhIL-37b) from *E.coli*, confirming molecular weights and immunospecificity by Coomassie Blue Staining and Western Blot (Fig. 5c). The biological activity of rhIL-37b wild type (WT) pro and mature forms were tested in murine RAW cells in vitro. The results show that both pro and mature forms of rhIL-37b suppress the IL-6 production in a dose-dependent manner under LPS stimulation (Fig. 5d). The RAW cells did not produce high levels of IL-1β with only LPS stimulation^38^. These data demonstrate that both pro and mature forms of IL-37b suppress IL-6 production in the murine RAW cell response.

To investigate the effects of different IL-37 variants on the suppressive function of IL-37b, we expressed and purified mature forms of human recombinant IL-37bV1, IL37bV2 and IL37bV1V2 to examine for potential differences in suppressive function using in vitro and in vivo models. Surprisingly, our in vitro results indicate that the mature WT and different variants are equally suppressive for IL-6 production in murine macrophage-derived RAW cells (Fig. 5e). The dose–response characteristics of the 3 mature IL-37 variants were undistinguishable from the wild type. We confirm the effects of different variants on the suppressive function of IL-37b using human primary cells (PBMC) after LPS stimulation. Our data show that the mature form of WT and different variants of IL-37b exhibit equal suppressive function for IL-6 production in gradient dependent manner in human PBMC (Fig. 5f). Overall, these data indicate that the mature form of IL37 WT, V1, V2 and V1V2 are equally suppressive of the IL-6 response in both the murine RAW cell and human PBMC response.

### rhIL37bWT and variants on murine periodontitis model

**Fig. 6:**
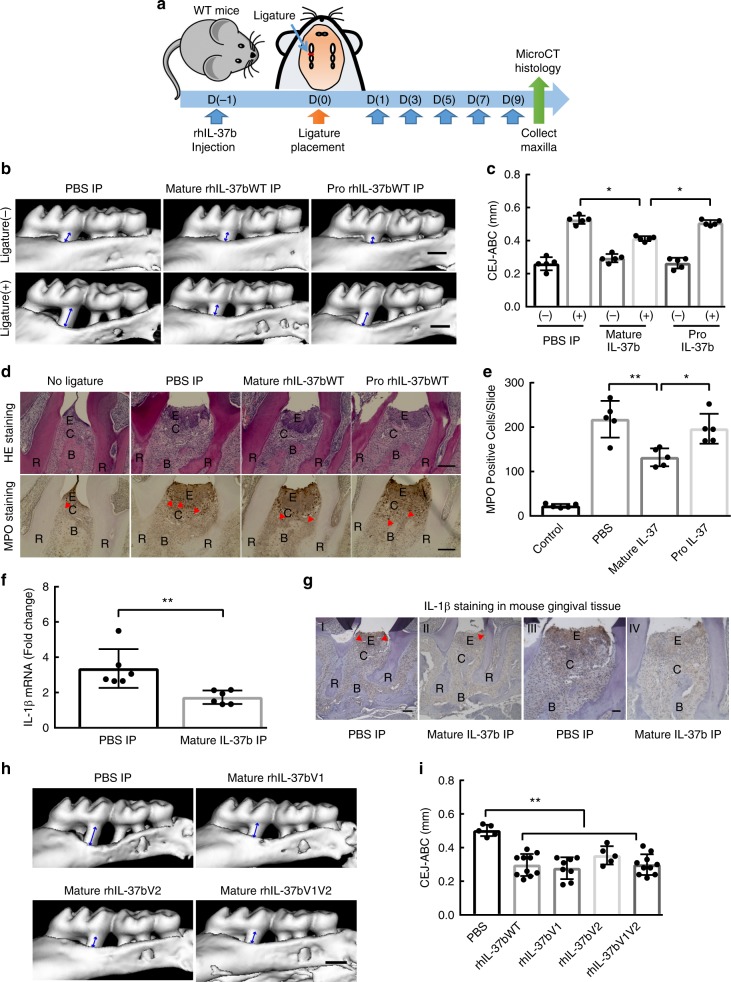
rhIL-37bWT and variants suppress the pathogenesis of periodontitis model in vivo. **a** The sketch to test the role of human recombinant IL-37b in murine periodontitis model. **b** Representative three-dimensional maxillary molars of pro, mature IL-37b and PBS injection groups. The distances from the cemento-enamel junction and alveolar bone crest (CEJ–ABC) of first molar root in buccal side of no-ligature and ligature sites are indicated by blue arrows. Scale bar is 0.5 mm. **c** The quantitative data of the distance from CEJ-ABC of first molar root in buccal side for each group. The results are expressed as the mean ± SD (*n* = 5/group). *p < 0.05 (One-way ANOVA and Tukey multiple comparisons tests). **d** Representative HE and MPO staining of pro, mature IL-37b and PBS injection groups. Scale bar is 200um. MPO positive cell are indicated by red arrowheads. E Epithelial layer. C Connective tissue. R Root of teeth. B Alveolar bone. **e** The quantitative data of the MPO positive cells between the root of first and second molar for each group. The results are expressed as the mean ± SD (*n* = 5/group). *p < 0.05, **p < 0.01 (One-way ANOVA and Tukey multiple comparisons tests). **f** The IL-1β expression level in the gingival tissues of ligature induced periodontitis model between PBS and mature IL-37bWT injection groups. The results are expressed as the mean ± SD (n = 6/group). **p < 0.01 (unpaired two tailed Student’s *t* test). **g** Representative IL-1β staining of PBS and mature IL-37b injection groups. IL-1β positive cell are indicated by red arrowheads. E (Epithelial layer). C (Connective tissue). R (Root of teeth). B (Alveolar bone). Scale bar is 200 μm for I and II. 500 μm for III and IV. **h** Representative three-dimensional maxillary molars of IL-37b V1, V2, V1V2, and PBS injection groups. Scale bar is 0.5 mm. **i** The quantitative data of the distance from CEJ-ABC of first molar root in buccal side for each group. The results are expressed as the mean ± SD (n ≥ 5/group). **p < 0.01 (One-way ANOVA and Tukey multiple comparisons tests)

We further investigate the in vivo effects of rhIL-37 in periodontitis development using a murine ligature-induced periodontitis model (Fig. 6a). In this model, the placement of ligatures between 1st and 2nd molars results in significant bone loss as compared to non-ligated teeth. Alveolar bone loss was evaluated by micro-CT in the ligated and non-ligated sides following an intraperitoneal (IP) injection with mature rhIL-37b or the pro-rhIL-37b (Fig. 6b, c). The results demonstrated that the mature form of rhIL-37b could significantly reduce alveolar bone loss as compared with the PBS control group. Interestingly, the pro form of rhIL-37b did not significantly suppress bone loss in vivo (Fig. 6b, c) via an IP administration. This suggests that the suppressive activity of pro-rhIL37 did not result in localized inhibition of alveolar bone resorption when provided as a distant, IP administration. We next histologically quantitated the inflammatory infiltrate in the gingival tissues by Myeloperoxidase (MPO) staining. Compared to the PBS injection group, our data show a significantly decreased MPO positive infiltration in the mature IL-37b injection group, but not the pro-rhIL-37 group (Fig. 6d, e). This histological data is in concordance with the alveolar bone loss data and confirm that the mature form of rhIL-37b inhibits immune cell infiltration and alveolar bone loss during the development of experimental periodontitis in this model. We also compared the gingival tissue IL-1β level between PBS and mature WT rhIL-37b IP group by qRCR and IHC. Our data indicated both the mRNA and protein level of IL-1b was significantly decreased in the gingival tissues of rhIL-37b IP group in the ligature induced periodontitis model (Fig. 6f, g). This data suggest that rhIL-37b could suppress IL-1β level in the inflamed gingival tissue.

In addition, the in vivo data also indicated the dramatic suppressive effects of alveolar bone loss was equal comparing the three types of all mature IL-37b variants following IP injection (Fig. 6h, i) in the bone resorption model. These data indicated that the IL-37 variants in the coding region of the mature IL-37b did not sufficiently impact the structure of the molecule to alter the suppressive function of the IL-37b protein in the bone resorption model. However, the findings clearly indicate that the activation of the pro form of IL-37 appears important for in vivo activity. Thus, we focused our experiments to further assess the modulation of IL-37 variants on the synthesis, cleavage and secretion of active mature IL-37.

### In vitro Caspase-1 binding and cleavage of rhIL-37bV1

Under normal inflammasome activation Caspase-1 activates IL-37 both intracellularly and extracellularly^39^. We examined the relative affinity for the rhIL-37V1 which has the two polymorphisms closest to the cleavage site of caspase-1. The cleavage of pro form to mature form from 0 to 30 min was determined by densiometric gel tracings and confirmed by western blot. A Lineweaver-Burk plot was generated measuring initial velocities at different substrate concentrations computed using rhIL-37WT as compared to rhIL-37V1 from Western blots (Supplementary Fig. 5). The data suggests that the rhIL-37V1 has lower binding affinity for caspase-1 as reflected in differences in intercept in the double-reciprocal plot. These results were only suggestive of potential differences in proIL-37V1 cleavage, but prompted us to explore in vivo IL-37V1 synthesis and activation more closely.

### mRNA and protein expression among WT and variants IL-37b

**Fig. 7:**
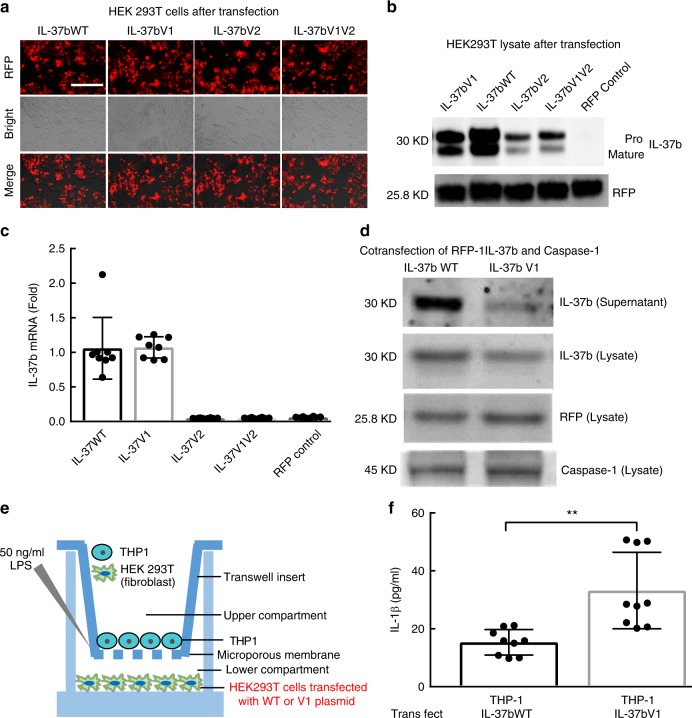
IL-37b expression in HEK293T cells transfected with IL-37bWT and Variants. **a** Representative picture of RFP positive HEK293T cells after transfection. Scale bar is 400 µm. **b** Western blot to detect IL-37b production in the cell lysates of HEK293T cells transient transfected with IL-37b WT and Variants plasmid. **c** IL-37b mRNA level in the cell lysates of HEK293T cells transient transfected with IL-37b WT and Variants plasmid. **d** IL-37b production in the supernatant and lysate of HEK293T cells co-transfected IL-37b (WT or V1) and caspase-1 plasmid. **e** The sketch of Transwell system to detect the suppressive activity of IL-37b WT and V1. **f** After LPS stimulation, IL-1β expression level in the supernatant of co-culturing THP-1 cells with HEK293T cells transfected by IL-37b WT or V1 plasmid. The results are expressed as the mean ± SD. **p < 0.01 (unpaired two-tailed Student’s *t*-test). Results are representative of three independent experiments

Given that all of the IL37 variants did not significantly alter the function of the mature IL-37b protein under the conditions tested, we examined whether the variants have an impact on the expression, secretion and activation of IL-37b. To test this underlying mechanism, we established the plasmids which can constitutively express WT or one of the three Variants of IL-37b and transiently transfected into human embryonic kidney (HEK293T) cells (Fig. 7a). Similar levels of transfection for plasmids were assured by using a RFP control. We compared the expression level of IL-37b WT protein and different variants via Western blotting in HEK293T cell lysates. Notably, we found that the protein level of IL-37b V1, IL-37bV2 and V1V2 appeared lower than IL-37bWT, in transfected HEK293T cell lysates, under unstimulated conditions (Fig. 7b). To test the potential possibility whether V1, V2 and V1V2 have effects on transcriptional expression level, we also performed qPCR to detect mRNA level. These data indicate that transfection of IL37V1 did not impact the mRNA expression of V1 as compared to WT; but showed a dramatically lower mRNA level in V2 and V1V2 as compared with WT and V1 (Fig. 7c). This suggests that the V2 and V1V2 may impact the instability of mRNA leading to small amounts of translation and protein production.

To examine the effects of the V1 SNPs on the cleavage of the pro form of IL-37b, we co-transfected the HEK293T cells with capsase-1 and measured the release of the mature protein into the supernatant. Under conditions in which we dually transfect the IL37V1 or IL37WT with caspase-1 to enhance processing, such that no pro-IL37 form is detectible, there is a significant decrease in mature IL-37V1 in the supernatant as demonstrated in the Western blots (Fig. 7d). This is supportive of our in vitro caspase-1 binding data (Supplementary Fig. 5). This suggests that IL-37V1 shows inadequate intracellular production and extracellular secretion. To confirm the altered biological activity associated with the IL37V1, we co-cultured the stably transfected HEK 293T cells (with WT or V1 plasmid) with LPS-stimulated THP-1 cells (Fig. 7e). Under these conditions we observe a statistically significant increase in IL-1β in the supernatant of IL37V1 as compared to IL37WT, as determined by ELISA (Fig. 7f). This represents a two-fold increase in IL-1β release.

### IL-37b expression and periodontitis model in transgenic mice

**Fig. 8:**
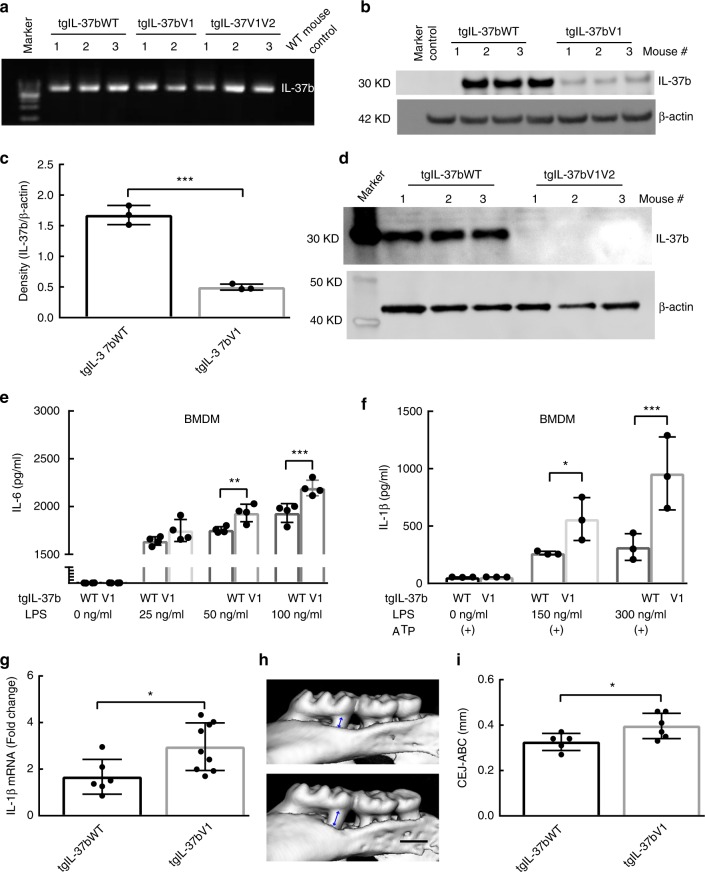
IL-37b expression among IL-37bWT and Variants in IL-37b transgenic mice. **a** The agarose gel confirms the *IL-37b* insert in IL-37WT, V1 and V1V2 transgenic mice which is absent in the background (control) WT strain. **b** IL-37b production in the blood cell lysates of tgIL-37WT, tgIL-37V1 and control mice. **c** Semi-quantitative band density analysis of IL-37b from panel b normalized to β-actin. Data are means ± SD (*n* = 3/group). ***p < 0.001(unpaired two-tailed Student’s *t*-test). **d** IL-37b production in the blood cell lysates of tgIL-37WT and tgIL-37V1V2 mice (*n* = 3/group). **e** IL-6 expression level in the supernatant of BMDM (Bone marrow-derived macrophage) from tgIL-37WT and tgIL-37V1 mice at various LPS concentrations. The results are expressed as the mean ± SD. **p < 0.01, ***p < 0.001 (One-way ANOVA and Sidak multiple comparisons test). Results are representative of three independent experiments. **f** BMDM IL-1β expression by tgIL-37WT and tgIL-37V1 mice at various LPS concentrations in presence of ATP. The results are expressed as the mean ± SD. *p < 0.05, ***p < 0.001 (One-way ANOVA and Sidak multiple comparisons test). (+) indicates the ATP stimulation for BMDM. Results are representative of three independent experiments. **g** The IL-1β expression level in the gingival tissues of ligature induced periodontitis model between IL-37WT and IL-37V1 transgenic mice group. The results are expressed as the mean ± SD (*n* ≥ 6/group). *p < 0.05 (unpaired two tailed Student’s *t* test). **h** Representative three-dimensional maxillary molars of IL-37WT and V1 transgenic mice in ligature induced periodontitis model. Scale bar is 0.5 mm. **i** The quantitative data of the distance from CEJ-ABC of first and second molar root in buccal side for each group. The results are expressed as the mean ± SD (*n* ≥ 5/group). (unpaired two tailed Student’s *t* test). *p < 0.05

Transgenic mice were created using the constitutively expressed IL-37b or variants under CAG promoter driving IL37 within the Rosa26 locus (Fig. 8a). There is strong constitutive production of IL-37 in unstimulated blood samples of the tgIL-37WT mice (Fig. 8b). There is a statistically significant diminished level of IL-37 among the tgIL37V1 (Fig. 8b, c) and no detectible blood levels of IL-37 in the tgIL37V1V2 mice (Fig. 8d). This transgenic mice data confirm the decreased production of IL-37 in the presence of the V1 and V1V2 variants in the HEK293T system. After stimulation of the bone marrow derived macrophages (BMDM) from tgIL-37WT and tgIL-37V1 mice with *E. coli* LPS (Fig. 8e), BMDM from tgIL-37V1 showed significantly higher IL-6 production compared with BMDM from tgIL-37WT (Fig. 8e). In addition, we demonstrate that the IL-1β secretion into the BMDM supernatant is significantly higher in the presence of the IL37V1, as compared to IL37WT under LPS and ATP stimulation (Fig. 6f). Again this is a difference of 2–3 fold enhanced secretion of IL-1β attributable to the variant 1 of IL37. In total, these experiments provide additional evidence that IL37V1 and IL37V1V2 are both associated with impaired production of IL-37, which in turn, results in an impaired suppression of IL-6 and IL-1β secretion.

To further investigate the periodontitis pathogenesis in vivo, we performed ligature-induced periodontitis model using IL-37WT and V1 transgenic mice and evaluated the alveolar bone loss and IL-1β level in the gingival tissue. Our data show that the IL-1β level in the gingival tissue is higher in the tgIL-37V1 mice than tgIL-37WT mice (Fig. 6g). In addition, the alveolar bone loss in the tgIL-37V1 mice is significantly higher than tgIL-37WT mice (Fig. 6h, i). These in vivo data further confirm that V1 SNP is associated with the pathogenesis of periodontitis including alveolar bone loss and IL-1β expression.

## Discussion

We have identified two missense polymorphic variants in the anti-inflammatory *IL37* gene locus that appear to be responsible for high GCF IL-1β levels. The discovery of these two *IL37* variants is significant, because IL-37 is a key suppressor of innate immunity and a master regulator of inflammation at mucosal surfaces^40^. IL-37 protein production has been shown to be upregulated in human dendritic cells when stimulated by TLR ligands^20,40,41^. In humans, blocking normal IL-37 expression results in a 2.1-fold increase in monocytic IL-1β expression in response to endotoxin challenge^40^. We also confirm the increased mRNA expression of IL-1β ιn LPS-stimulated dendritic cells isolated from homozygous *IL37V1* subjects compared to IL37WT (Fig. 3b). These findings indicate that the IL-37 function is diminished in the presence of the SNP variants. Interestingly, the previously reported effect of the IL-1β SNP at rs16944 as enhancing IL-1β synthesis^42^ is also confirmed in our study. Overall, these findings demonstrate the strong association between *IL37 SNP and GCF IL-1*β levels.

This study discovered IL-37 using GCF IL-1β levels as the targeted quantitative trait. It has been previously reported that IL-37 could downregulate multiple pro-inflammatory cytokine production^14,43^. Therefore, it is plausible to hypothesize that the other inflammatory cytokines highly involved in the pathogenesis of periodontitis^44^ could also be influenced by IL-37. Measuring the multiplex cytokines levels using GCF samples collected from IL-37b WT and V1 suggest that V1 is associated with higher periodontal GCF levels of IL-1β and IL-8.

The IL-37V1 locus is associated with severe chronic periodontal disease in the Dental ARIC population. The potential association of aggressive periodontitis in the Dental ARIC population cannot be ascertained, as this is an older cohort where many additional teeth might be expected to have been lost. However, the association with severe attachment loss at baseline examination followed by a 10-year loss of 3 or more teeth is certainly consistent with the key role of this *IL37* QTL as a modulator of inflammation and disease expression. Although IL-37V2 appears to be associated with higher levels of GCF-IL1-β, this does not appear unless there are one more copies of the IL-37V1, so the independent contribution of IL-37V2 to periodontal status or tooth loss in the Dental ARIC population could not be ascertained.

The identification of *IL37* using GCF IL-1β levels vs serum levels is likely a reflection of the direct, local gingival tissue activation of the inflammatory response to the adjacent microbial biofilm, as compared to the systemic, whole-body interactions that modulate serum IL-1β levels. The direct GCF sample avoids whole body adsorption and metabolism to provide a distinct advantage for measuring inflammatory molecules. This report also provides the population-based evidence that the IL-37V1 is associated with not only more severe periodontal disease and tooth loss but also stroke prevalence. Although these associations were not genome-wide significant and need to be replicated and validated in other populations, they provide a context for appreciating the potential significance of the genetic variance of this important anti-inflammatory master cytokine in human disease.

In diseased periodontal tissues of unspecified genotype, IL-37 is widely expressed within the epithelium and plasma cells (Fig. 4). IL-37 production has not been previously reported in plasma cells and due to the dominance of plasma cells in the periodontal lesion, these cells may play an important role in periodontal disease pathogenesis and merit further investigation. Furthermore, Isoform 1 appears to dominate within the tissues. Isoform 4 is also present, but has been predicted to not have biological activity^45^, however, this has not been experimentally confirmed and needs further study. In addition, although the location of the exonic SNPs of V1 and V2 are not in proximity to splice regions, this may be one plausible explanation for alternate splicing isoforms.

During normal function, IL-37 is cleaved intracellularly by caspase-1 and translocated extracellularly where it activates IL-18 receptor signaling pathways^46^ or to the nucleus with SMAD3 interaction to suppress multiple signaling pathways^47^. In the murine periodontitis model, IL-37 inhibits bone loss and inflammatory cell infiltrate (Fig. 6). This blockage of bone loss represents this biological function for IL-37 and suggests that IL-37 agonists might be a therapeutic target for preventing bone loss in inflammatory conditions, including periodontal disease. However, whether the mechanism by which IL-37 inhibits bone loss are dependent on IL-18 receptor and SMAD3 needs further investigation.

In summary, the scientific merit of the current investigation hinges upon the central importance of understanding the genetic basis of the immune response in humans. Unraveling the genetic basis of pathogenesis will lead to improved diagnoses and prognoses by identifying individuals at risk for disease to optimize prevention strategies^48–52^. For example, subjects identified early in life to be at risk with one or more copies of the *IL37* risk allele could receive more frequent preventive maintenance treatments in order to prevent the onset of periodontal disease. Other mucosal conditions such as inflammatory bowel disease, which is associated with high gut IL-1β production and partly regulated by IL-37^53^, may also be modulated by this risk allele. Furthermore, up to 30% of the Caucasian population may have this genetic trait that would ultimately enable the design of pharmacological treatment strategies directed to restoring IL-37 function and reducing inflammation in a precisely targeted population.

## Methods

### Study cohorts

IRB approval consistent with the Helsinki guidelines was obtained for all study cohorts used in these analyses. The Dental ARIC and the functional studies of IL-37 were both approved by the Institutional Review Board (IRB) of the University of North Carolina at Chapel Hill. The Dutch and German Study of Aggressive Periodontitis were approved by the ethics commission of the medical faculty of the Christian-Albrechts-Universität zu Kiel, Germany. All enrolled subjects signed informed consent after receiving an oral and written description of the informed consent.

### Measurement of IL-1β in GCF

GCF was collected from the mesio-buccal site of each of the four first molar teeth, one in each quadrant^54^. Briefly, samples were frozen chairside in liquid nitrogen and stored in liquid nitrogen until processing. Four GCF strips were eluted and analyzed separately for each of the 4910 subjects who also had genotype data. IL-1β levels were evaluated by enzyme-linked immune-absorbent assay (ELISA) according to the manufacturer’s instructions as described^55^. GCF analyte concentration data were pooled to provide a person-level mean value in ng/ml. An additional serum sample from each subject was assayed for serum IL-1β levels in duplicate and expressed as a mean value for each subject. GCF levels of IL-1β, IL-6, IL-8, TNFα, G-CSG and MIP1β were measured by immunobead multiplexing for a random sample of genotyped subjects in the DARIC population (*n* = 107) and supplemented with an additional 36 subjects who were genotyped for the *IL37V1* locus by pyrosequencing to enrich the population for the minor allele. Levels of each mediator were normalized (z-score transformed) to the 1.1 allele of IL37V1 and tested using linear mixed models overall, as well as pairwise post-hoc.

Demographic characteristics of the analytical sample were presented and contrasted according to high and low GCF-IL-1β categories using X^2^ or t-tests. The association of clinical disease traits with the *IL37V1* polymorphism were tested using logistic regression models to compute odds ratios and 95% confidence intervals adjusting for examination center, gender, age, diabetes, smoking (5-level) and the first 10 ancestry principal components. Associations of clinical disease traits and *IL37V1* polymorphism with 10-year tooth loss were tested using Poisson regression with robust error variance to compute relative risks. Association of clinical traits as continuous variables by each *IL37V1* haplotype were tested by GLM in SAS 9.4.

### Genotyping, imputation and quality control

Blood collection from an antecubital vein and DNA extraction using PUREGENE® DNA Purification Kit were performed at a central ARIC laboratory in Houston, TX^56^. The Affymetrix 6.0 chip was used for genotyping. Quality control procedures and SNP imputation was performed using HapMap Phase II CEU build 36^57^. Estimates of relatedness and population stratification in ARIC was done as described^56^. The final genotyping was performed on a subset of 85947 “high quality” SNPs with MAF ≥ 0.1, a call rate > 99.5%, and satisfied Hardy-Weinberg equilibrium at *P* ≥ 10^−3^. First and second degree relatives were identified and excluded, and ancestry was controlled using EIGENSTRAT computing 10 principal component, as previously described^58^.

### Analytical strategy

The association between individual SNPs and the log-transformed GCF-IL-1β levels was tested using linear regression models assuming additive allelic effects, adjusting for age, sex, examination center and ancestry (10 first ancestral PCs). The association with GCF-IL-1b was also tested using quartiles and upper 90th percentiles of the IL-1 QTL using logistic regression, adjusting as above. We report the beta coefficients with standard errors and P values for each trait. We applied a genome-wide significance level of P < 5 × 10^−8^ (correcting for multiple-testing) using the ProbABEL software^59^. LocusZoom ver.4.8^60^ was used to view genomic regions of interest. For the IL-1 response trait we generated Q-Q plots and Manhattan plots.Custom programs^56^ were used to generate *lambda* coefficients. Ensembl Variation Database v75 and PolyPhen-2 were used for the prediction of coding, non-synonymous potentially damaging protein structural changes^61^.

Online resources included the UCSF Genome browser v274 at (http://genome.ucsc.edu/). Tools available at the National Center for Biotechnology Information (NCBI- http://www.ncbi.nlm.nih.gov/) and we used the “HUGO Gene Nomenclature” naming convention (http://www.genenames.org) for gene reporting. Individual loci were annotated using 1000 genomes, when possible. The rs identifiers, minor allelic frequencies and p values are presented on-line as Supplementary Data 1.

Stepwise linear models for log-mean GCF-IL-1β levels stratified by high GCF-IL-1β upper quartile and low GCF-IL1β (lower three quartiles) were performed, not for hypothesis testing, but to identify those SNPs associated with the biphasic distribution of IL-1 responses within normal (Q1–3) and hyper-inflammatory levels (Q4) of IL-1β. SAS v9.4 was used to identify significant SNPs with r squared, F and P values for each of the distributions.

The estimation of the proportion of variance of IL1β using the GWAS SNPs was done using Genome-Wide Complex Trait Analysis (GCTA)^62^. A pairwise genetic relationship matrix was constructed using the autosomal genotypes of the ARIC participants. This matrix with restricted maximum likelihood is used to estimate the variance proportion explained by the SNPs with a minor allele frequency greater than 5% (656,292 SNPs). Covariates of examination center, sex and age and 10 principal components were also included in the analysis.

### Pyrosequencing for human SNP Identification

The flanking sequences of our SNP of interest (rs3811046 and rs3811047) were obtained through Ensembl search (https://useast.ensembl.org/index.html). Because the two SNPs were only 31 nucleotides apart, primers were designed to amplify both rs3811046 and rs3811047 regions. Assay Design Software (BiotageAB, version 1.0.6) was used to design the forward and reverse primers, as well as a sequencing primer (forward primer: TGCTAACCTCACTGCGTCTGAC, biotin labeled at 5′ end; reverse primer: ATCACCTCACCCCGAGGC; sequencing primer: CCTTACTTGTGTGAACAAA). The forward and reverse primers were used to amplify (Hotstar Taq polymerase, QIAgen. Hilden, Germany) our region of interest by the DNA purified (using PT-L2P kit from DNA genotek. Ontario, Canada) from the saliva (collected through an Oragene.DNA saliva DNA kit from DNA genotek. Ontario, Canada) of the participants. Sequencing primer was used for downstream sequencing through a pyrosequencer (PyromarkMD from QIAGEN. Hilden, Germany). Based on the genotype results, the participants were classified as 1.1 (homozygous for the major alleles), 1.2 (heterozygous for the major and minor alleles), or 2.2 (homozygous for the minor alleles). The known genotyped participants were contacted, and when the participants showed interest in, and consented for, further participation, appointments were made for peripheral blood draw via venipuncture.

### Human monocyte isolation and dendritic cell differentiation

Approximately 50 mL of whole blood was diluted in phosphate buffered saline with 2 mM EDTA into 300 ml. The diluted blood was layered over Ficoll-Paque PLUS (GE Healthcare. Illinois, USA) and centrifuged to isolate peripheral blood mononuclear cells (PBMC). After lysing away any remnants of erythrocytes and removing platelets, CD14 microbeads were used to isolate monocytes through positive selection by magnetic cell sorting (Miltenyi Biotech. Bergisch Gladbach, Germany). The isolated monocytes were plated in a 24-well plate, ~0.7–1 × 10^6^ cells per well in 600 μL of RPMI with 100 U/mL penicillin, 100 μg/mL streptomycin, with 10% fetal bovine serum in each wells. Final concentrations of 500 U/mL of IL-4 (Peprotech. New Jersey, US) and 1000 U/mL of GM-SCF (Peprotech. New Jersey, US) were added to the media for 2 h after initial plating and at 3 days when media was changed. At 7 days, cells was changed into media without IL-4 and GM-CSF and continue culturing  2 h before stimulation. *E. coli* (strain O111:B4) LPS was added to final concentration of 0.1 μg/mL. The stimulated cells were harvested at 0, 1, 6, 12, and 24-h time points. mRNA was extracted using RNeasy Mini Kit (QIAGEN. Hilden, Germany). IL-1β expression levels (Hs01555410_m1) were measured and compared between WT homozyogote and V1 homozygote groups. GAPDH (Hs03929097_g1) was used as internal control for ∆∆Ct calculation.

### *IL37* variants in German cohort of aggressive periodontitis

The study population of German and Dutch AgP cases and controls have been genotyped and imputed^63^. The independent German and Dutch GWAS data were meta-analyzed using a fixed-effects model because the two study samples showed a high level of homogeneity for rs3811046, *p*-value of Cochran’s QP (Q) > 0.05 and heterogeneity index *I*^2^ < 0.5.

### mRNA Extraction and IL-37 Quantitative RT-PCR

Human gingival biopsies were taken from subjects with healthy periodontium and chronic periodontal disease according to the AAP/ADA classification^64^. All enrolled participants into this study provided written informed consent, which was approved by the Institutional Review Board (IRB) of the University of North Carolina at Chapel Hill (IRB number, 15–0335). Upon removal, total RNA was isolated from these human gingival tissue biopsies using the Qiagen RNA extraction kit and cDNA was synthesized using Vilo according to the manufacturer’s instructions. Expression level of 5 different isoform of IL-37 were measured by qPCR using the ABI 7500 real-time RCR system with SYBR green (Applied Biosystems). Isoform specific primers listed below were designed by flanking the junction of exon and confirmed by Agarose gel. Isoform1: Forward: 5-TCACACAAGTCCAAAGGTGA-3, Reverse: 5- AGCCAGCTTCATCAGTTTCT-3. Isoform2: Forward: 5-GCTTAGAAGGTCCAAAGGTGAA-3, Reverse: 5- GAGCTCAAGGATGAGGCTAATG-3. Isoform3: Forward: 5- GCTGCTTAGAAGAGATCTTCTTT-3, Reverse: 5- CTGAAGGGATGGATGACTTTG-3. Isoform4: Forward: 5-TCACACAAAGATCTTCTTTGCA-3, 5-CAGCCAGCTTCATCAGTTTC-3. Isoform5: Forward: 5-GCGCTTAAGAGGTCCAAAGGT-3, 5-GCTATGAGATTCCCAGAGTCCAG-3.

### Recombinant human IL-37b expression and purification

Pre (1–218 amino acids) and mature (21–218 amino acids) forms of human IL-37b (WT and Variants) were cloned in pET-28C and confirmed by sequencing. Recombinant human IL-37b (rHIL-37b) was expressed in *E.coli* BL21 (DE3) and purified through Ni-NTA agarose gel column. Purified proteins were identified by Commassie Blue staining and Western Blot. Identified recombinant human IL-37b (rhIL-37b) was freeze-dried by lyophilization and kept in −20C for further in vitro and in vivo utilization.

### In vitro recombinant IL-37b suppression assay

RAW cells (ATCC TIB-71^TM^) were seeded into 96-well plates with 5 × 10^4^/well in DMEM with 10% FBS and cultured overnight. Human PBMC were purified from whole blood samples by Ficoll-Paque PLUS (GE Healthcare. Illinois, USA) and seeded into 96-well plates with 1 × 10^5^ in RMPI RPMI 1460 containing 10% FBS, 2 mM glutamine, 1% nonessential amino acids, 1% sodium pyruvate, and 50 U/ml penicillin and streptomycin. After overnight culture, these cells were pretreated with gradient concentrations of recombinant IL-37b from 300 ng/ml to 0.3 ng/ml for 24 h. Then, the cells were changed into fresh culture medium with same concentration of recombinant IL-37b following adding 50 ng/ml E.coli LPS. After 8 h LPS stimulation, culture supernatants were collected and level of IL-6 in the supernatant of RAW cells were determined by DuoSet ELISA kit (DY406, R&D System) and level of IL-6 in the supernatant of human PBMC were determined by DuoSet ELISA kit (DY206, R&D System).

### Animals

Female Specific Pathogen Free (SPF) mice, 8–10 week old C57B6/Ntac wild-type (WT) were purchased from Taconic Farms (USA). WT and Variants of IL-37b transgenic mice were generated by UNC Animal Models Core facility to be expressed from the strong CAGG promoter in the ROSA26 locus and confirmed by sequencing. The transgenic mice were genotyped by PCR with the following primers: IL-37b: Forward: 5-TCACACAAGTCCAAAGGTGA-3, Reverse: 5-AGCCAGCTTCATCAGTTTCT-3. Rosa: Forward: 5-TAAGGGAGCTGCAGTGGAGTA-3, 5-TTTAAGCCTGCCCAGAAGACT-3. Correctly genotyped transgenic female mice (8–10 weeks old) were used for all murine experiments. All mice were housed under specific pathogen-free conditions at the animal facility of the University of North Carolina. Mice were given regular mouse chow diet and water ad libitum with a cycle of 12 h of light and 12 h of darkness. All of the animal studies followed the IACUC standards for the use and care of animals and were approved by the animal ethics committee from the University of North Carolina.

### Ligature induced periodontitis model and rhIL-37b treatment

SPF mice were randomly allocated into different experimental groups and murine periodontitis was induced by ligature installation between first and second molars^65^. Briefly, silk sutures (Roboz surgical store, SUT-15–1, Gaithersburg, MD) were inserted between the first and second maxillary molars of isoflurane-anesthetized mice. Suture string ends were knotted to prevent ligature loss. The silk sutures were kept between the molars for 10 days to induce periodontitis. Recombinant human IL-37b (1ug/mouse) and same volume of vehicle control (PBS) were given intraperitoneally to mice 6 times during the process of ligature induced periodontitis model (−1, 1, 3, 5, 7, and 9 days with ligature installation). Mice maxillae were harvested after euthanasia following fixation with 10% formalin for MicroCT and histological analysis in different groups.

### Immunohistology of human gingival biopsies and mice maxilla

Human gingival tissues were also fixed in 10% neutral buffered formalin overnight at room temperature, dehydrated in 70% alcohol, and then embedded in paraffin for the immunohistochemical procedure. Gingival tissue sections (5μm thick) were obtained in the sagittal direction including the epithelial layer and connective tissues. The slides were stained with anti-IL-37 antibody with 1:1000 dilution (rabbit anti-human, ThermoFisher SCIENTIFIC, PA5-30527), anti-CD138 with 1:150 dilution (goat anti-human CD138, R&D SYSTEMS, AF2780) antibody. Anti-rabbit and goat HRP-DAB staining was used according to the manufacturer’s instructions (R&D SYSTEMS, CTS008 and CTS005) and the slides were counterstained with hematoxylin. Co-localization of IL-37 with CD138, CD38 (rabbit anti human, MyBioSource; MBS302125), Cytokeratin 5 (Mouse anti human, Millipore Sigma; MAB1620) or CD11c (Mouse anti human, Novus Biologicals; NBP2-44599) was detected with Cy5-conjugated Donkey Anti-Rabbit (Jackson ImmunoResearch; 711-175-152), Alexa Fluor 488-conjugated Donkey Anti-Goat (Jackson ImmunoResearch; 705-545-147) or Alexa Fluor 488-conjugated Goat Anti-Mouse (Abcam; ab150113) of secondary antibody. The dilution of IL-37, CD138, CD38, Cytokeratin 5 and CD11c antibody for co-localization is 1:500, 1:75, 1:50, 1:100 and 1:200 times, respectively. The dilution for fluoresces-conjugated secondary antibody is 1:500. Nuclei were visualized by DAPI staining according to the manufacturer’s instructions. Photo images for DAB staining were captured using an Olympus BX61 microscope and co-localization of fluorescence staining were captured using a Zeiss LSM 700 confocal microscope.

Mice maxilla samples were decalcified with 10% EDTA, embedded and sliced with 5μm-thick sections. Sections were sliced in the sagittal direction along the long axis of the teeth and included the distal root of the first molar and the mesio-root of second molar. The sections were stained with hematoxylin and eosin, myeloperoxidase (MPO, R&D SYSTEMS, AF3667) and IL1β (Abcam; ab9722). The dilution for MPO and IL-1β antibody is 1:200 and 1:100 times. MPO positive cells were counted at the gingival tissues between the distal root of the first molar and the mesio-root of second molar using ImageJ software. Mice gingival tissues around all three molars were collected from both ligature and no-ligature sides for RNA extraction. IL-1β expression levels (Mm00434228_m1) were measured and compared between groups. GAPDH (Mm99999915_g1) was used as internal control for ∆∆Ct calculation. The groups are blinding to performer for the MPO counting and IL-1β qPCR detection.

### MicroCT scanning and analysis

Formalin-fixed maxillae were scanned in all three spatial planes at a resolution of 18 μm with a micro CT 100 micro-computed tomography system (Scanco Medical, Brüttisellen, Switzerland)). To detect the alveolar bone loss, the distance between the cementoenamel junction (CEJ) and alveolar bone crest (ABC) was measured at one sites for the first molars (disto-buccal) three-dimensional images viewed from the buccal sides^65^. The measurements were repeated two times per site, and mean distances were presented in millimeters. The groups are blinding to performer for the microCT measurement.

### RFP plasmid of IL-37b and HEK293T transfection bioassay

IL-37b WT, V1, V2, V1V2 were cloned into EF.CMV.RFP (addgene Plasmid #17619) downstream of the EF1a promoter and right clone were selected by sequencing. Constructed plasmids were transfected into HEK293T cells (ATCC CRL-3216^TM^) using Lipofectamine 2000 (Invitrogen). RFP positive cells were observed under fluoresce microscope. After 24 h transfection, cell lysate were collected for Western Blot analysis. IL-37b WT and V1 EF.CMV.RFP plasmids were also co-transfected with Caspase-1 plasmid into HEK293T cells; culture supernatant and cell lysate were collected for testing the interference of Variants to Casepase-1 cleavage of IL-37 by ELISA and Western Blot. IL-37b WT and V1 EF.CMV.RFP plasmids were also co-cultured with THP-1 (ATCC TIB-202^TM^) cells for 24 h, then 50 ng/ml LPS were added into co-culture system to stimulate THP-1 cells purchased from ATCC for 8 h. Culture supernatant were collected to detect IL-1β level by ELISA (DY201, R&D system).

### Western blot

Cells were lysed with RIPA buffer containing phosphatase and proteinase inhibitors and were loaded into 4–12% gradient SDS-PAGE gel (Thermo Fisher Scientific). The proteins were transferred into a 0.2 μm nitrocellulose membrane which were blocked with 5% skim milk in phosphate buffered saline solution (PBS) containing 0.1% Tween-20. Primary antibody was applied overnight at 4 °C. The membranes were washed 2 times for 5 min each with 0.1% Tween-20 containing PBS (PBST), then incubated with horseradish peroxidase (HRP) conjugated secondary antibody for 45 min. After washing twice again with PBST, the immunoblots were visualized using ImageQuant LAS4000 luminescent image reader (GE Healthcare Life Sciences, USA). Anti-human IL-37 polyclonal goat antibody (AF1975, Novus biologicals, 1:200 dilution), HRP conjugated anti-RFP polyclonal rabbit antibody (PM005-7, MBL Life Science, 1:2000 dilution), Anti Myc-Tag monoclonal rabbit antibody (# 2278 s, Cell Signaling, 1:3000 dilution) anti-beta actin polyclonal goat antibody (Ab8229,Abcam, 1:500 dilution). Donkey anti goat (Santa Cruz, SC-2020), Goat anti rabbit (Jackson Immuno research 111-035-144) and Goat anti mouse (Promega W 402B) HRP conjugated secondary antibodies were used against the corresponding primary antibodies with a 1:10,000 times dilution. The uncropped scans of Western blots used the above antibody were also provided (Supplementary Fig. 6–8).

### Statistical analysis for cell and animal studies

Differences between two groups were evaluated using unpaired two tailed Student’s *t*-test. Multiple group comparisons were performed using one-way ANOVA, and then the different types of post-hoc multiple comparisons tests. Differences at *p < 0.05 were considered significant. Statistical analyses were performed using SAS v9.4 or GraphPad Prism software.

## Electronic supplementary material

Supplementary Information

Peer Reviewer File

Description of Additional Supplementary Files

Supplementary Data 1

## Data Availability

The entire GWAS results appear as Supplementary Data 1 and are additionally available at http://genomewide.net/public/aric/dental/gcf_il1b/IL1_trait.txt. All other relevant data are available within the main text and its Supplementary Information or from the corresponding authors upon reasonable request.
