# Supplementary material for: GWAS for Interleukin-1β levels in gingival crevicular fluid identifies IL37 variants in periodontal inflammation
Source: Nat Commun. 2018 Sep 11;9:3686. doi: 10.1038/s41467-018-05940-9 (PMC6134146; doi:10.1038/s41467-018-05940-9)
Supplement: Supplementary file 1 — Supplementary Information [file 41467_2018_5940_MOESM1_ESM.pdf]

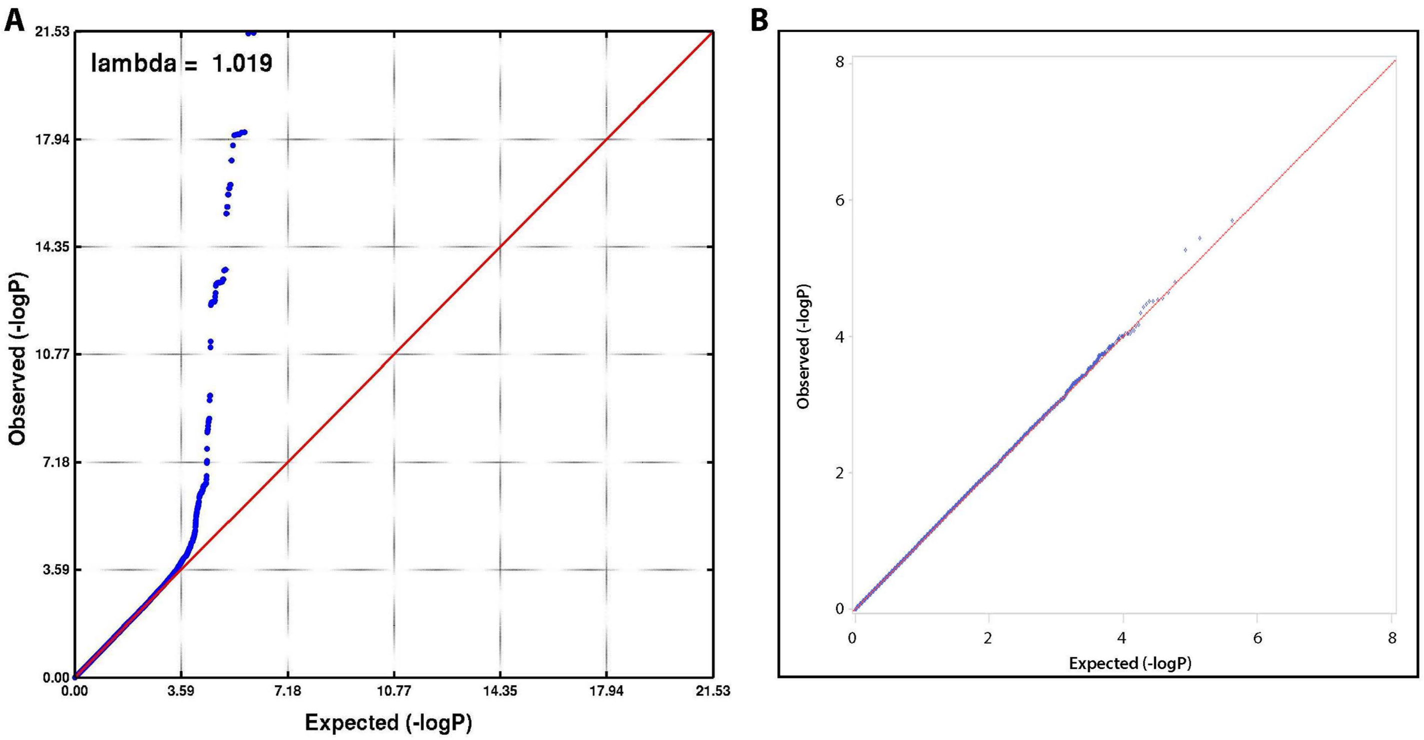

Supplementary Figure 1: QQ Plot for Top Quartile of GCF IL-1β.
There was virtually no genomic inflation without significant confounding of the variance of GCF IL-1β level.

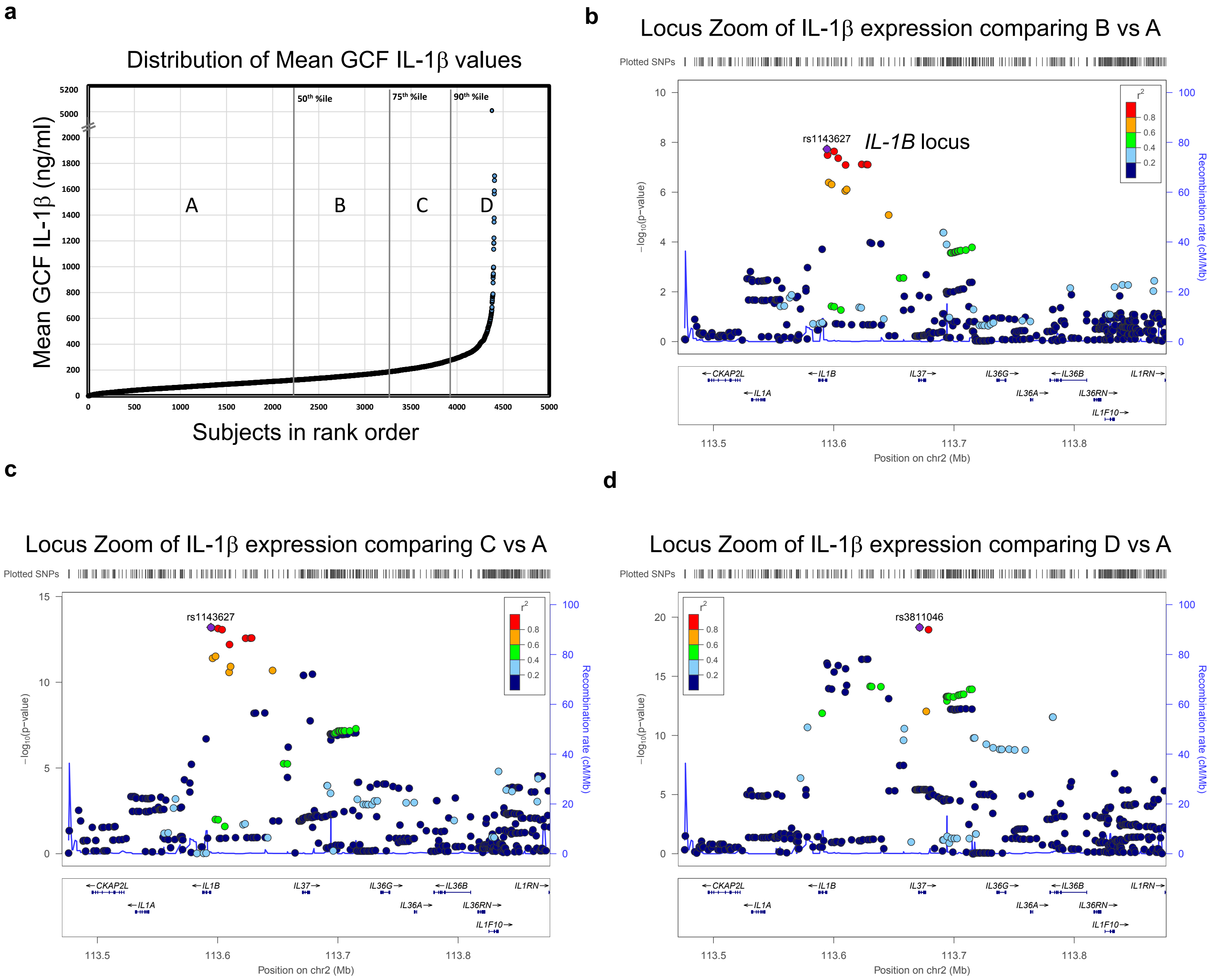

Beta (p-value) for each GWAS run

	BvsA	CvsA	DvsA
rs16944	-0.33 (3.24e-08)	-0.56 (6.54e-14)	-0.76 (1.09e-16)
rs3811046	0.15 (1.39e-02)	0.46 (3.96e-11)	0.74 (7.25e-20)

Supplementary Figure 2: (a): Distribution of GCF IL-1β levels, IL-1β values among subjects and there is a marked inflection beginning near the 75th percentile. (b): LocusZoom comparing people within the 50-75th percentiles vs 0-50th percentiles, (c): LocusZoom comparing people within the 75-90th percentiles vs 0-50th percentiles. (d): LocusZoom comparing people within the 90-100th percentiles vs 0-50th percentiles.

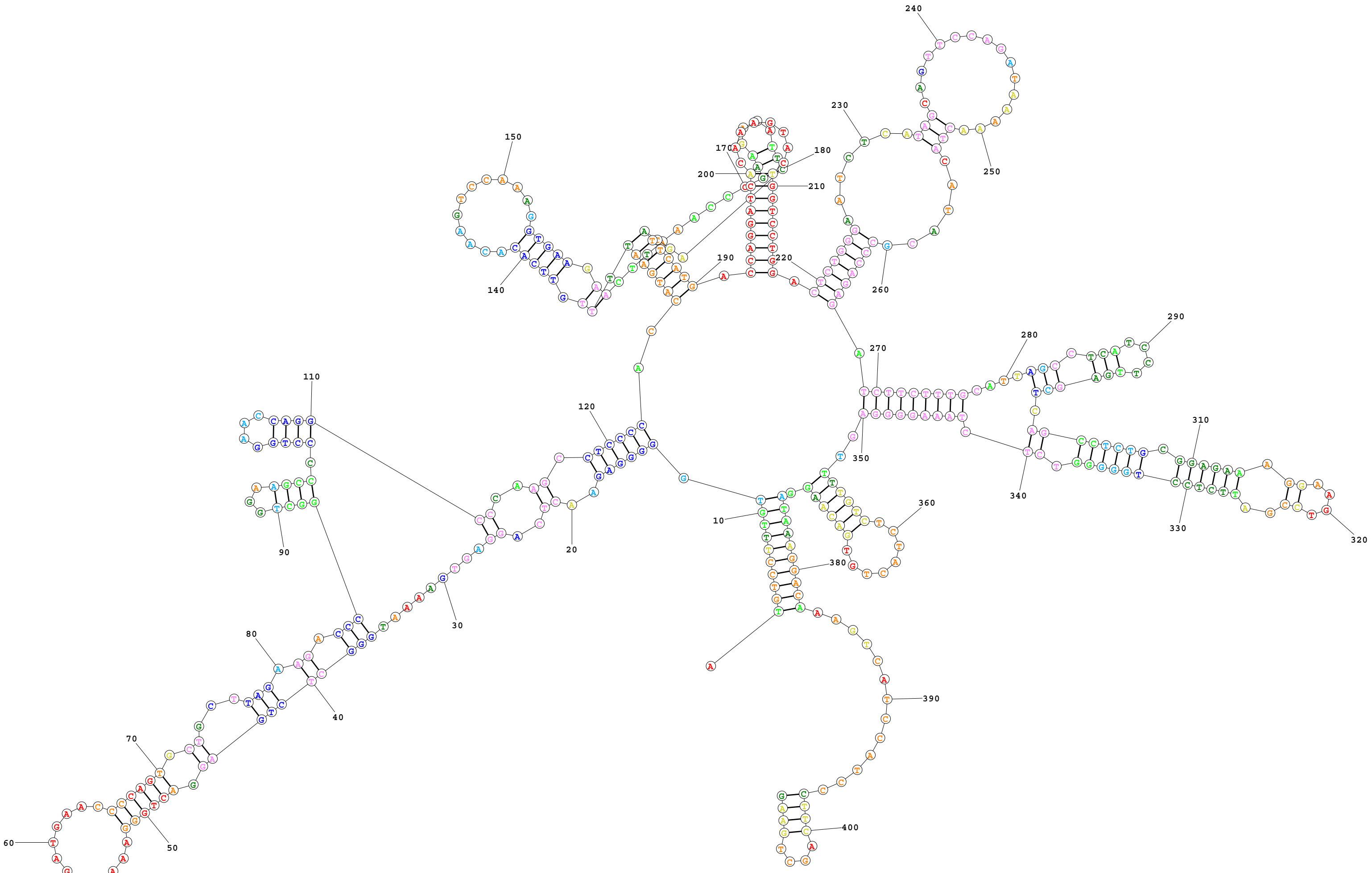

Supplementary Figure 3: mRNA structure prediction of IL-37bWT.

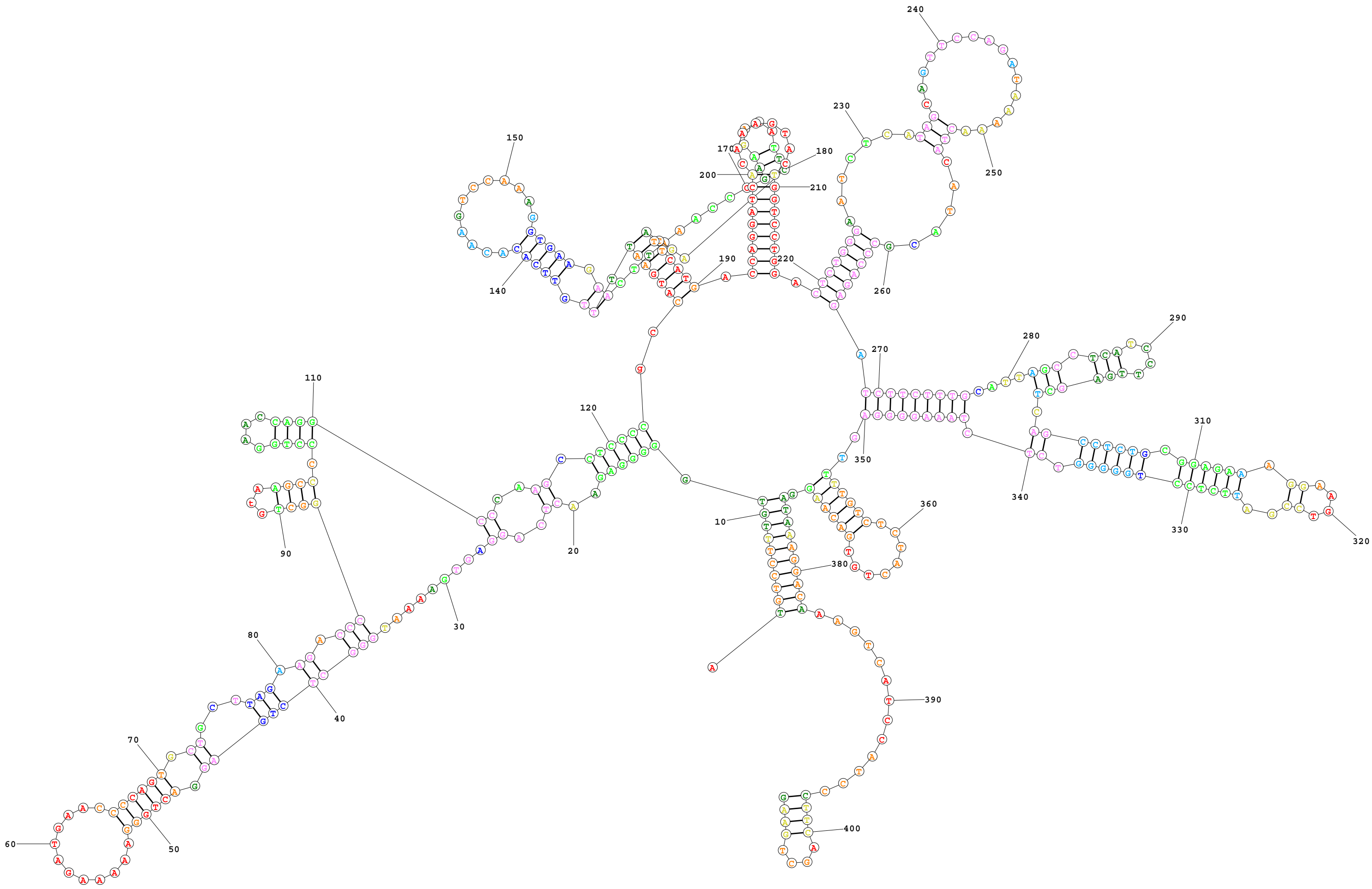

Supplementary Figure 3: mRNA structure prediction of IL-37bV1.

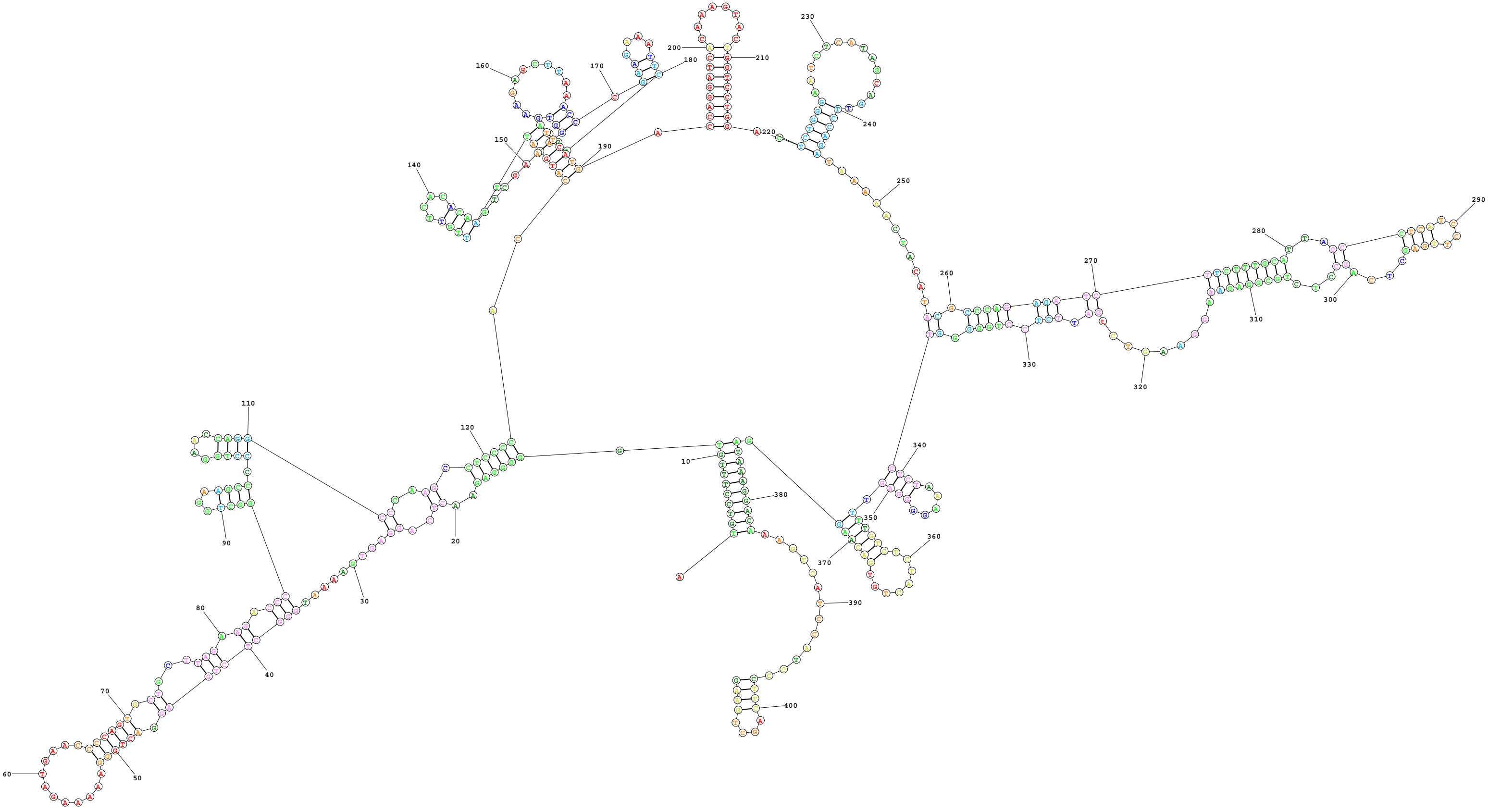

Supplementary Figure 3: mRNA structure prediction of IL-37bV2.

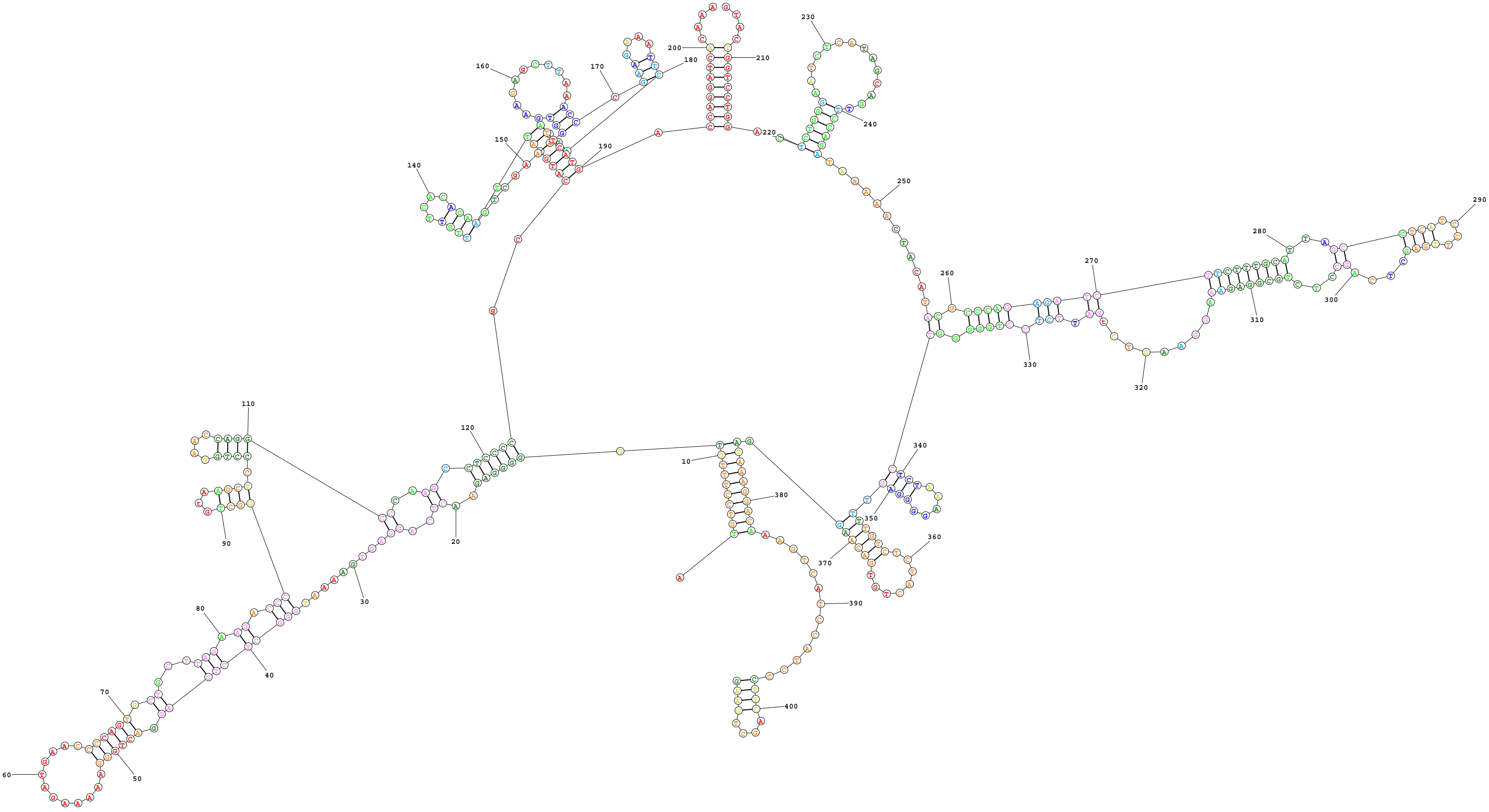

Supplementary Figure 3: mRNA structure prediction of IL-37bV1V2.

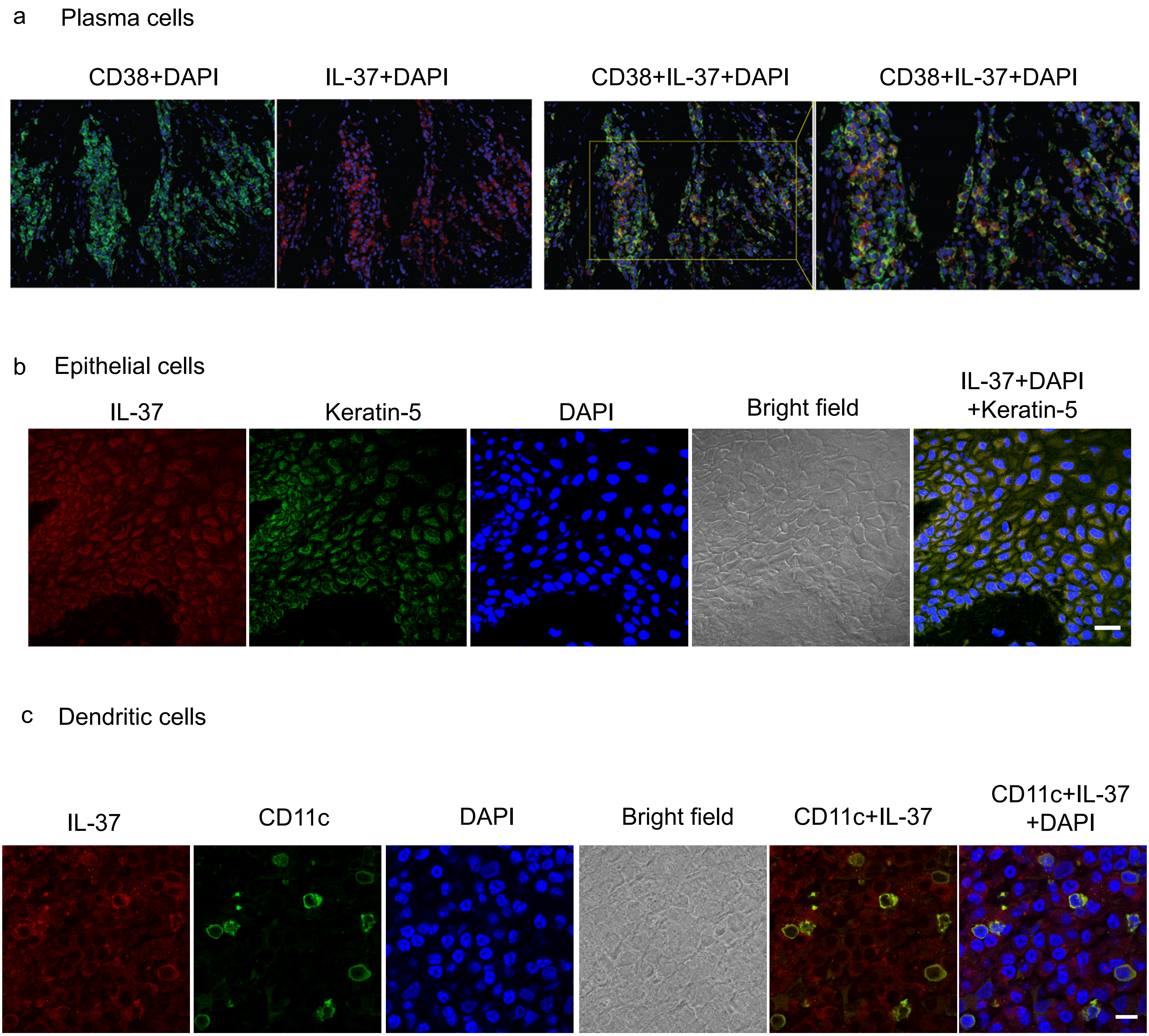

Supplemental Figure 4: (a) Co-localization of IL-37 and CD38 in human gingival tissue.
(b) Co-localization of IL-37 and Keratin-5 in human gingival tissue.
(c) Co-localization of IL-37 and CD11c in human gingival tissue. Scale bar is 10um.

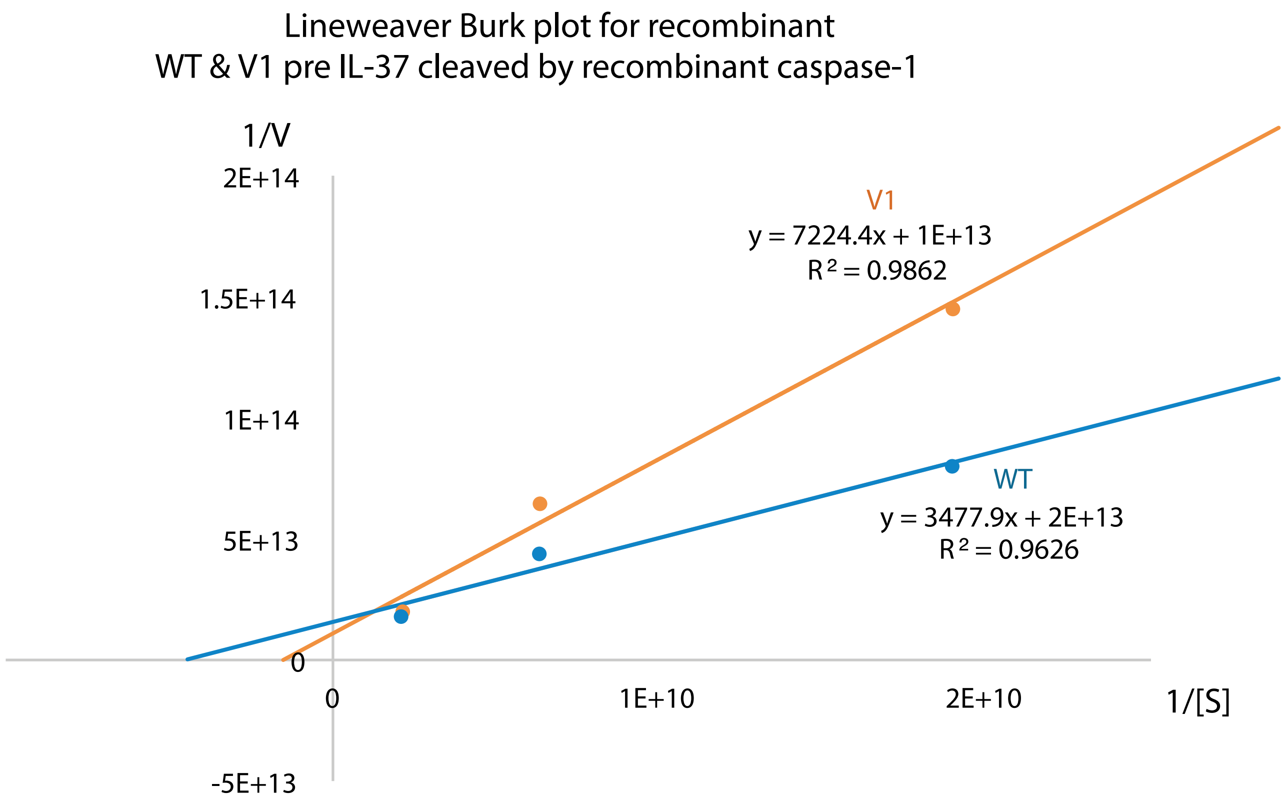

Supplementary Figure 5: Lineweaver-Burk plot to measure the initial velocities at different substrate concentrations computed using rHIL-37WT as compared to rHIL-37V1.

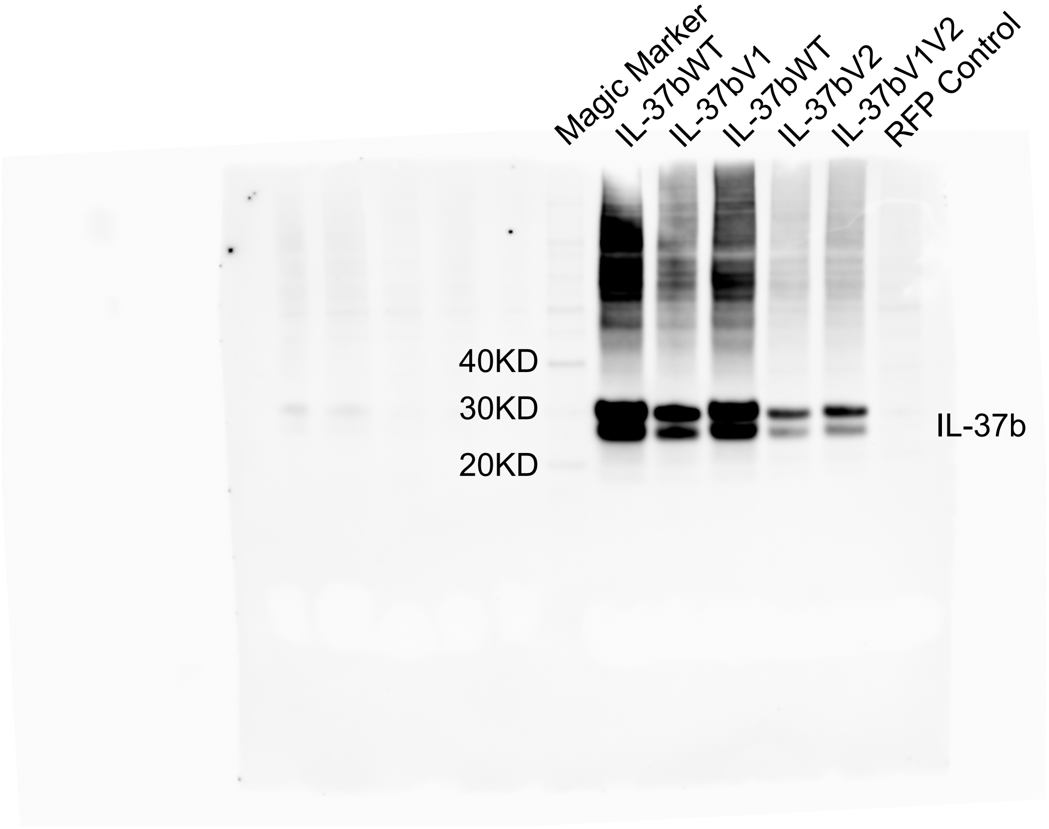

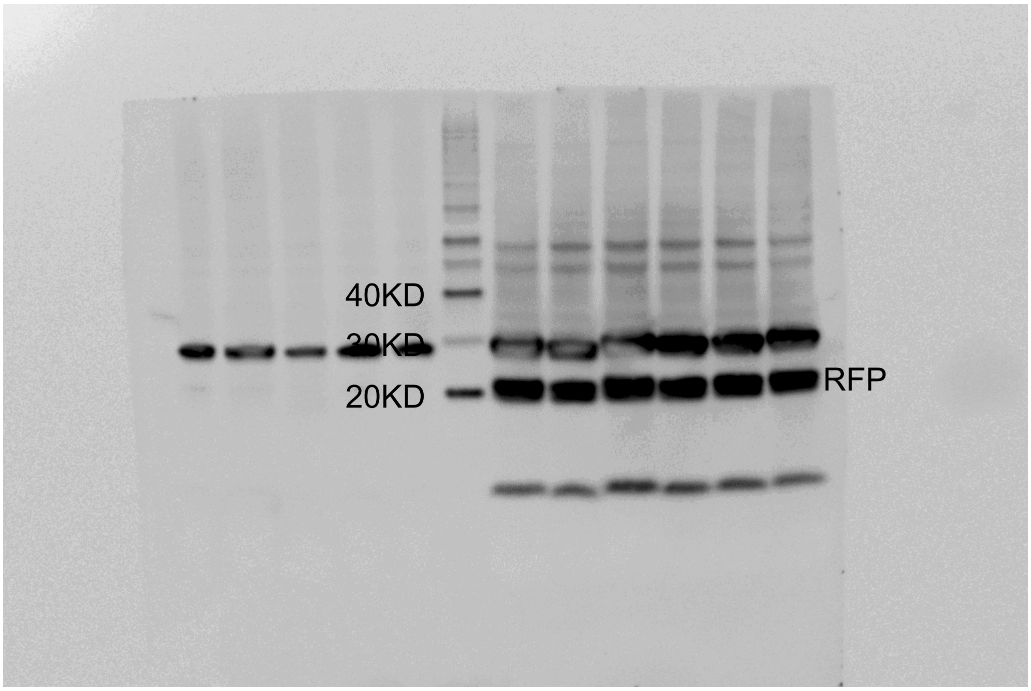

Supplementary Figure 6: The uncroped gel blot for Figure 7b.

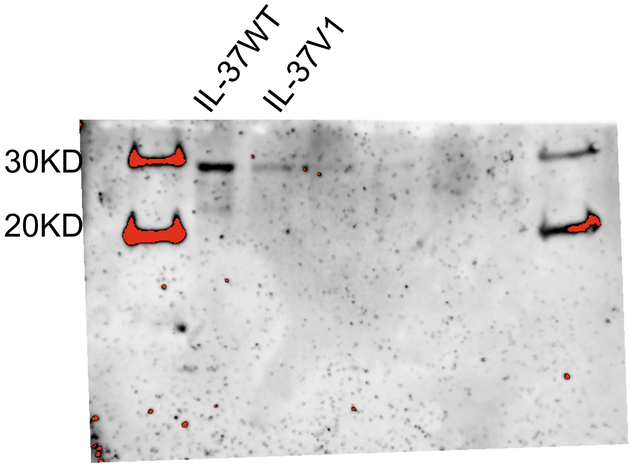

Supernatant

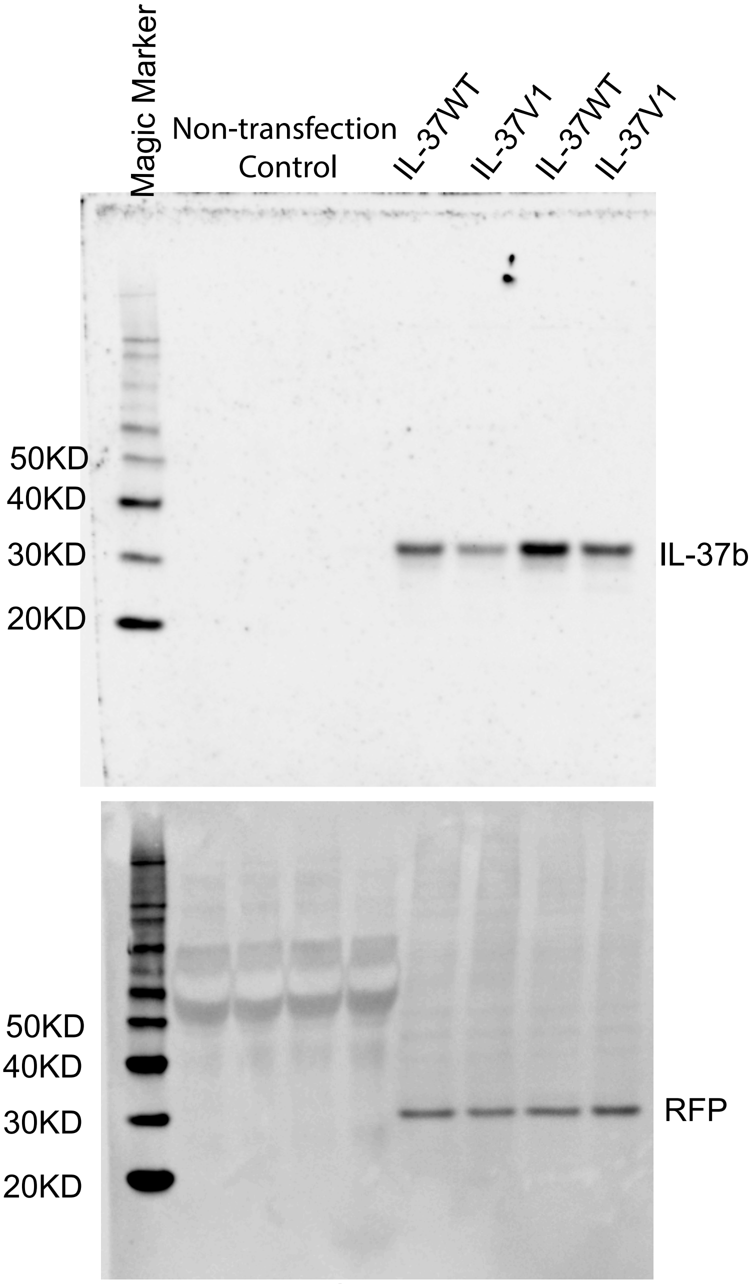

Lysate

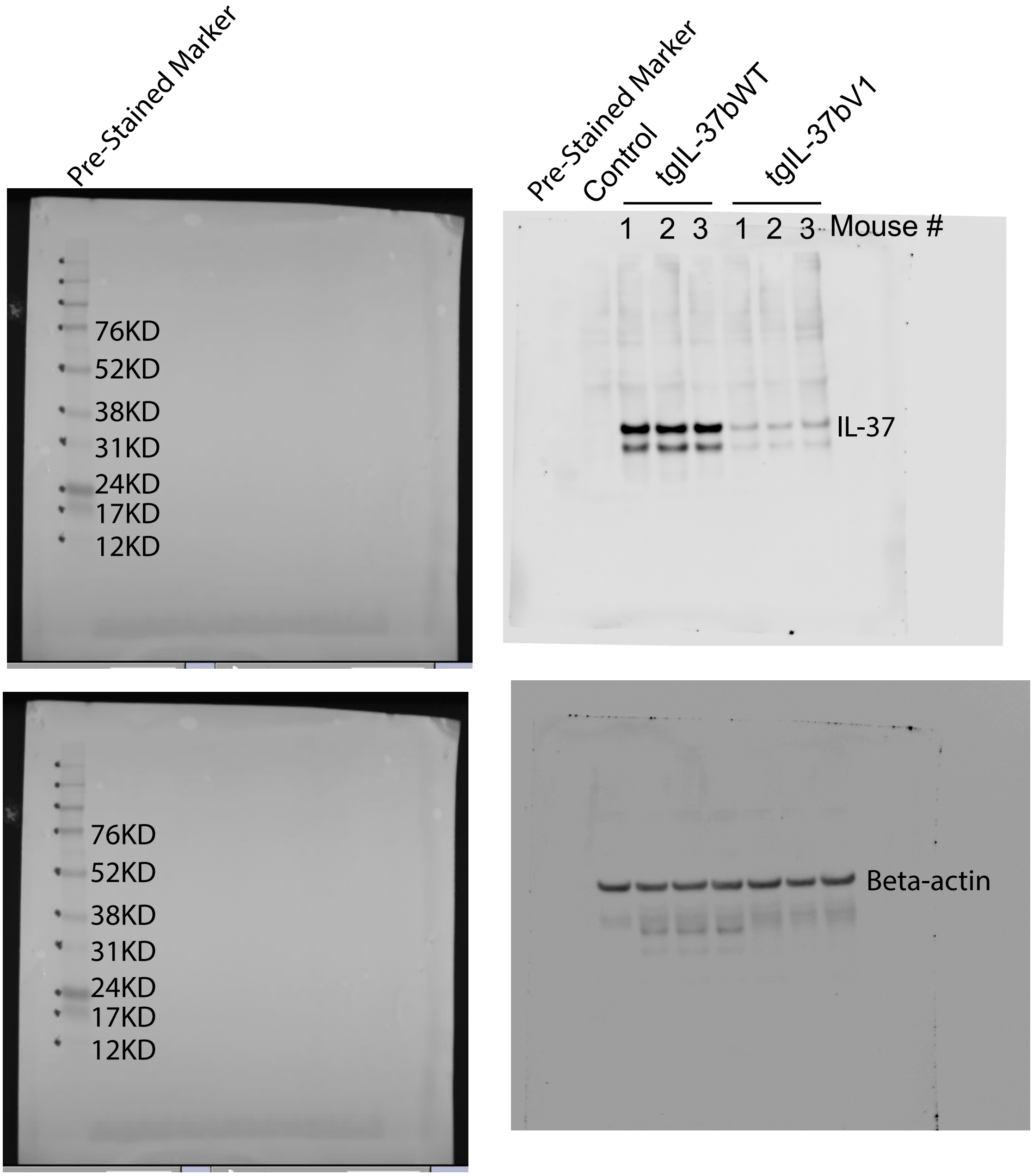

Supplementary Figure 8: The uncroped gel blot for Figure 8b.

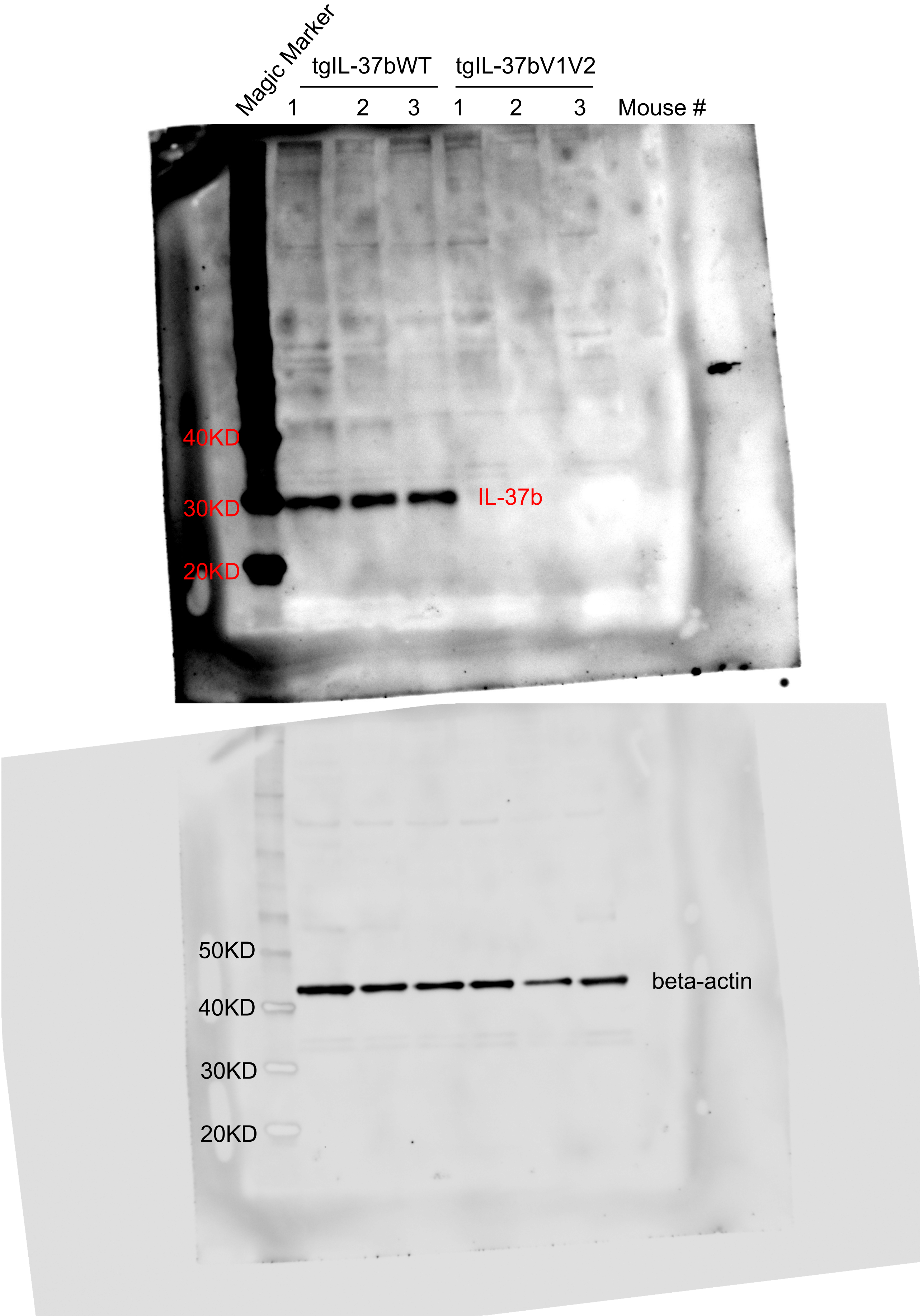

Supplementary Figure 9: The uncroped gel blot for Figure 8d.

Supplementary Table 1. Characteristics of Dental ARIC population according to GCF IL-1β Levels.

	**Entire sample (n=4,408)**	**"Low" GCF IL-1β (0-75th Percentile) (n=3320)**	**"High" GCF IL-1β (>75th Percentile) (n=1088)**	**p-value (X^2 test, ANOVA)**
Age [years; mean (SD)]	62.7 (5.60)	62.6 (5.59)	62.9 (5.60)	0.19
Gender				0.11
Male	2107 (47.8%)	1564 (74.2%)	543 (25.8%)	
Female	2301 (52.2%)	1756 (76.3%)	545 (23.7%)	
Diabetes diagnosis				0.0004
Diabetic	499 (11.3%)	344 (68.9%)	155 (31.1%)	
Non-Diabetic	3903 (88.7%)	2972 (76.2%)	931 (23.9%)	
Carotid Intima-media thickness				0.002
≥1mm	718 (26.5%)	528 (73.5%)	190 (26.5%)	
<1mm	5386 (88.2%)	4241 (78.7%)	1145 (21.3%)	
Examination Center				0.73
NC	1306 (29.6%)	994 (76.1%)	312 (23.9%)	
MN	1855 (42.1%)	1392 (75.0%)	463 (25.0%)	
MD	1247 (28.3%)	934 (74.9%)	313 (25.1%)	
Body Mass Index [mean (SD)]	28.1 (5.03)	28.0 (4.98)	28.5 (5.16)	0.007
Smoking status				<0.0001
Never Smoker	1997 (46.3%)	1542 (77.2%)	455 (22.8%)	
Former Smoker, Light	962 (22.3%)	739 (76.8%)	223 (23.2%)	
Former Smoker, Heavy	825 (19.1%)	620 (75.2%)	205 (24.9%)	
Current Smoker, Light	77 (1.8%)	50 (64.9%)	27 (35.1%)	
Current Smoker, Heavy	451 (10.5%)	297 (65.9%)	154 (34.2%)	
Periodontal Profile Class				<0.0001[a]
PPC Health	1727 (27.8%)	1406 (82.4%)	321 (18.6%)	Ref[b]
PPC Mild Perio.	956 (15.4%)	709 (74.2%)	247 (25.8%)	<0.0001
PPC High Gingival Index	644 (10.4%)	563 (87.4%)	81 (12.6%)	0.0006
PPC Moderate Perio.	698 (11.3%)	548 (78.5%)	150 (21.5%)	0.10
PPC Tooth loss	918 (14.8%)	655 (72.4%)	263 (28.7%)	<0.0001
PPC Severe Tooth Loss	824 (13.3%)	651 (79.0%)	173 (21.0%)	0.15
PPC Severe Perio.	440 (7.1%)	323 (73.4%)	117 (26.6%)	0.0002
CP diagnosis (CDC/AAP)				<0.0001 [a]
Health	512 (11.8%)	403 (78.7%)	109 (21.3%)	Ref[b]
Mild	1261 (20.0%)	972 (77.1%)	289 (22.9%)	0.95
Moderate	174 (41.9%)	1394 (76.6%)	426 (23.4%)	0.36
Severe	753 (17.3%)	509 (67.6%)	244 (32.4%)	<0.00001
BGI periodontal diagnosis				<0.0001 [a]
BGI Health	536 (12.3%)	442 (82.5%)	94 (17.5%)	Ref[b]
G1	585 (13.5%)	428 (73.2%)	157 (26.8%)	<0.0001
P1	899 (20.7%)	722 (80.3%)	177 (19.7%)	0.01
P2	1866 (42.9%)	1403 (75.2%)	463 (24.8%)	<0.0001
P3	461 (10.6%)	284 (61.6%)	177 (38.4%)	<0.0001

[a] Overall P value "Chunk test" [b] Pairwise comparisons using Health as referant.
BGI: Biofilm-gingival interface CP: Chronic Periodontitis
High IL-1β was also associated with more severe periodontal disease, using 3 different taxonomies: the PPC 7-level definition, the 4-level CDC/AAP definition and the BGI classification.

Supplementary Table 2. Stepwise Linear Models for Log Mean stratified on High and low GCF-IL1β.

Step	SNP Entered	Gene	Partial R-Square	Model R-Square	Pr > F
High GCF-IL1β Step1	rs3811046	*IL37*	0.0098	0.047	0.0015
High GCF-IL1β Step2	rs3811047	*IL37*	0.0075	0.0545	0.0051
Lower GCF-IL1β Step1	rs1143627	*IL1B*	0.0072	0.0135	<.0001
Lower GCF-IL1β Step2	rs16944	*IL1B*	0.0028	0.0163	0.0029
Lower GCF-IL1β Step3	rs13027999	*IL38*	0.0016	0.0179	0.0246
Lower GCF-IL1β Step4	rs13426809	*IL36G*	0.0012	0.0192	0.0472

High GCF-IL1β: n=1027, upper quartile
Low GCF-IL1β: n=3151, lower three quartiles
For this analysis, we included all SNPs with p values less than $5x10^{-6}$ in the models and adjusted for gender, age and ancestry (first 10 principal components).

Supplementary Table 3. Adjusted* Relative risk (and 95% confidence intervals) between rs3811046 genotype and prospectively assessed (incident) 10-year tooth loss (≥3 teeth lost) in the Dental ARIC sample (incidence proportion=12.4%, total sample n=2766).

**10-year Incident Tooth loss (≥3 teeth)**	**1.1 (TT)**	**1.2+2.2 (TG + GG)**
Main Effect Model		
rs3811046	Ref	1.33 (1.09-1.62)
Interaction Model (IL-37V1*PPC)		
PPC-A [Health]	Ref	2.07 (1.18-3.63)
PPC-B [Mild disease]	2.51 (1.40-4.49)	3.02 (1.73-5.28)
PPC-C [High Gingival Index]	3.21 (1.41-7.32)	3.14 (1.37-7.23)
PPC-D [Tooth Loss]	4.73 (2.71-8.27)	6.47 (3.82-10.9)
PPC-E [Posterior Disease]	3.56 (2.07-6.12)	3.67 (2.13-6.32)
PPC-F [Severe tooth Loss]	2.56 (1.32-4.99)	3.88 (2.10-7.16)
PPC-G [Severe Disease]	5.27 (2.75-10.1)	5.75 (3.15-10.5)

*Adjusted for Examination Center, Gender, Age, Diabetes, Smoking (5-levels) and 10 Ancestry PCs. ARIC Cohort includes genotyped, dentate, Caucasian individuals that were available for 10-year follow-up questionnaire. It includes the Dental ARIC cohort subset that received dental examination data at baseline (Visit 4). PPC=Periodontal Profile Class. The change in BIC (Bayesian Information Coefficient) is 7.44 reflecting a "strong" improvement in the model by the addition of the SNP variant status.

Supplementary Table 4. Logistic regression model*-predicted probabilities of 10-year tooth loss (≥3 teeth) by rs3811046 genotype and chronic periodontal disease status.

		**rs3811046 (IL-37V1) genotype**		
Chronic Periodontal Disease Status	N	1.1(TT)	1.2(TG)	2.2(GG)
PPC-A [Health]	1461	0.05	0.06	0.08
PPC-B [Mild disease]	811	0.10	0.12	0.17
PPC-C [High Gingival Index]	158	0.13	0.15	0.18
PPC-D [Tooth Loss]	530	0.23	0.28	0.32
PPC-E [Posterior Disease]	827	0.13	0.16	0.21
PPC-F [Severe tooth Loss]	474	0.14	0.17	0.20
PPC-G [Severe Disease]	237	0.24	0.30	0.34

*Logistic regression model including terms for examination center, gender, age, diabetes, smoking (5 levels) and ancestry (10 PCs). In adjusted logistic models all strata demonstrated a greater positive predictive value for 10-year tooth loss with the carriage of the minor allelic variant, irrespective of periodontal status. Statistical significance was achieved for most comparisons with a clear allelic dose-response effect across all periodontal categories, including health.

Supplementary Table 5. Odds ratios and 95% confidence intervals for prevalent Stroke stratified on IL-37v1 genotype in the Dental ARIC sample (prevalence=1.6%, sample n=4464).

Odds Ratio Prevalent Stroke (95% CI)	rs3811046 WT-1.1(TT)	rs3811046 V1-1.1+1.2(TG + GG)
Overall	Ref	2.08 (1.25-3.46)
PPC-A [Health]	Ref	4.72 (1.32-16.8)
PPC-B [Mild disease]	1.55 (0.31-7.86)	6.13 (1.67-22.4)
PPC-C [High Gingival Index]	2.50 (0.25-25.6)	--
PPC-D [Tooth Loss]	2.58 (0.55-12.1)	1.66 (0.32-8.59)
PPC-E [Posterior Disease]	1.19 (0.23-6.08)	2.88 (0.72-11.6)
PPC-F [Severe tooth Loss]	4.70 (1.11-19.9)	5.79 (1.46-23.0)
PPC-G [Severe Disease]	5.66 (1.08-30.8)	8.53 (1.92-38.0)

Rs3811046 genotype: WT 1.1 TT vs V1 1.2+2.2 TG + GG. Adjusted for Gender, Age, Examination Center, Diabetes, Hypertension, Smoking (5-levels), HDL, Cholesterol, BMI and 10 Ancestry PCs. The change in BIC (Bayesian Information Coefficient) is 9.08 reflecting a “strong” improvement in the logistic model by the addition of the SNP.
