# Supplementary material for: GWAS for Interleukin-1β levels in gingival crevicular fluid identifies IL37 variants in periodontal inflammation
Source: Nat Commun. 2018 Sep 11;9:3686. doi: 10.1038/s41467-018-05940-9 (PMC6134146; doi:10.1038/s41467-018-05940-9)
Supplement: Supplementary file 3 — Description of Additional Supplementary Files [file 41467_2018_5940_MOESM3_ESM.pdf]

## Description of Supplementary Data files

*Supplementary Data 1*

GWAS results for GCF-IL-1beta upper quartile vs lower three quartiles
