# Supplementary material for: GWAS for Interleukin-1β levels in gingival crevicular fluid identifies IL37 variants in periodontal inflammation
Source: Nat Commun. 2018 Sep 11;9:3686. doi: 10.1038/s41467-018-05940-9 (PMC6134146; doi:10.1038/s41467-018-05940-9)
Supplement: Supplementary file 2 — Peer Reviewer File [file 41467_2018_5940_MOESM2_ESM.pdf]

Reviewer #1 (Remarks to the Author):

Offenbacher et al. report the association of an IL-37 polymorphism with high level of IL-1beta in gingival crevicular fluid in healthy and periodontitis patients. This report is important to understand the genetic background of the risk for periodontitis development, and the genetic analyses are well designed and the conclusions are driven from fine results with controls. However, the experiments following genetic analyses have dampened my enthusiasm, because IL-37 does not exist in rodents. Specific comments are shown below:

Specific comments:

1. The authors used mice and RAW cells to determine the functions of human IL-37 variants in the experiments of Figures 5 and 6. Because rodents do not have IL-37, it is important to stimulate human primary culture cells with the IL-37 molecules. This point is very critical for this manuscript.

2. The figure legends are minimal. It is important to provide more information to readers in the figure legends.

3. "We determined V1 genotypes for 143 individuals by pyrosequencing" (pp15): The method for "pyrosequencing" is missing.

Reviewer #2 (Remarks to the Author):

The authors describe two novel allelic variants of IL-37 associated with high levels of IL-1β and therefore increased inflammatory response and bad prognosis in periodontitis. They show that IL-37 genetic variants are unable to exert the anti-inflammatory activity of WT IL-37 and the consequent excessive IL-1β causes a more severe disease in patients and in an IL-37-transgenic mouse model. By dissecting the molecular mechanisms, they propose that the variants affect mRNA expression and protein activation by caspase-1 and secretion of active mature forms, whereas the functional activity of the variant proteins is not affected.

The findings are interesting and novel, and contribute in understanding the genetic basis of IL-1-dependent innate immune response as a determinant of inflammatory conditions in humans.

However, the manuscript is not easy to read and could be better presented. Some specific analysis could be better detailed.

More specifically:

- The first part, in particular Figures 1-3, is difficult to understand for a general public. Figure legends must be implemented and detailed to render figures more comprehensible. Define inset of Fig 1b.

-

- The specific cellular source of IL-37 in periodontitis should be better defined, using other markers than CD138 and CD38 (Figure 4 and Supplementary figure 4). The contribution of other cell types could be better examined.

The authors use dendritic cells from patients to analyse the effect of IL-37 variants. Are dendritic cells producing IL-37 and IL-37 target in periodontitis in vivo?

- The authors focused on IL-1β GCF levels to define genes associated with periodontitis severity, since it is considered a key prognostic factor in periodontitis. The contribution of other inflammatory cytokines involved in the pathogenesis (e.g. IL-17A) that could be influenced by the altered IL-37 function should be at least discussed. Figure 3A: the increased levels of the inflammatory mediators analysed should be discussed.

- A more detailed analysis of the mouse models (Figure 6 and Figure 8) would be interesting. Is there a difference in IL-1β level in mice treated with IL-37, in the model shown in figure 6? Importantly, the in vivo periodontitis model should be performed in V1IL-37 transgenic mice (Figure 8). Finally, is the immune infiltrate altered by IL-37 variants?

-

- The text language and manuscript organization could be improved.

- There are typos.

Reviewer #3 (Remarks to the Author):

This is a comprehensive body of work investigating genetic control of inflammation in periodontal disease. The findings implicate IL37 using both genomic and functional assays.

Comments:

1. In the abstract, it is not clear why different cut off values were selected to define high (quartiles) versus elevated IL-1B levels (percentiles / median).

2. Is IL37 expressed in cells from non-diseased periodontal tissue?

3. In the introduction, it would be helpful to add where the IL37 gene maps when it is first mentioned to make it clear that it is within the IL1 gene cluster on 2q13.

4. In table 1, adding the sample size of the entire cohort under the entire sample title would help.

5. Discovery GWAS section: the sentence beginning "The phenotypic variance..." does not make sense to me.

6. Was any conditional analysis performed to confirm that there are independent signals across the IL-1 complex locus?

7. I found Table 2 hard to interpret. I was expecting to see haploytpes but this seemed to be a diplotype analysis of genotype combinations. Haplotypes should be the alleles and would make it easier to interpret.

8. The manuscript could be shortened by avoiding repetition and with some editing eg in sections 'Associatoin of IL37 variants with prevalent...,aggresive periodontitis,....ARIC populations'.

9. I would like to see p-values in Table 6. I am concerned this data is being over-interpreted, given the wide confidence intervals for some groups. It detracts from the main message of the paper and I would advise moving it to supplementary material. I think it is also misleading to claim association in the discussion.

10. The Discussion is very long and sometimes a repetition of the results.

11. Have IL-1 genes been associated with CVD at genome-wide significance? Again, the section in the Discussion risks over-interpreting the findings.

Response to Reviewers' comments:

For Reviewer #1 (Remarks to the Author):

1: The authors used mice and RAW cells to determine the functions of human IL-37 variants in the experiments of Figures 5 and 6. Because rodents do not have IL-37, it is important to stimulate human primary culture cells with the IL-37 molecules. This point is very critical for this manuscript.
Response: We thank the reviewer for this critical suggestion. We have performed the additional experiment to test the role of rHIL-37 in human PBMC with the stimulation *of E.coli* LPS. Our data indicates that rhIL-37 WT as well as its variants also show the suppressive function of cytokine production (IL-6) by human PBMC. We have incorporated our new data that appears as Figure 5 f.

2. The figure legends are minimal. It is important to provide more information to readers in the figure legends.
Response: Thanks the reviewer's comment. We have revised and provided more information for the figure legends.

3. "We determined V1 genotypes for 143 individuals by pyrosequencing" (pp15): The method for "pyrosequencing" is missing.
Response: We have added our pyrosequencing protocol into materials and methods.

For Reviewer #2 (Remarks to the Author):

1: However, the manuscript is not easy to read and could be better presented. Some specific analysis could be better detailed.
Response: We appreciate this constructive suggestion from the reviewer. We have revised our manuscript for a better presentation and included more details in the text and in the legends.

2: The first part, in particular Figures 1-3, is difficult to understand for a general public. Figure legends must be implemented and detailed to render figures more comprehensible. Define inset of Figure 1b.
Response: This is a great point. We have implemented our figure legend according to reviewer's suggestion.

3: The specific cellular source of IL-37 in periodontitis should be better defined, using other markers than CD138 and CD38 (Figure 4 and Supplementary figure 4). The contribution of other cell types could be better examined.
Response: We really appreciate this reviewer's suggestion. From the IHC staining using DAB, we observed that plasma cells are the most robust infiltrated inflammatory cells in the diseased connective tissue. We therefore assess the co-localization of plasma cells marker (CD138 or CD38) and IL-37 in our first submission. We also found epithelial cells are the other major source of IL-37 expression in human gingival tissue biopsies. According to reviewer's suggestion, we also performed the immunofluorescent staining to confirm the co-localization of IL-37 with epithelial (Cytokeratin 5) in this resubmission. These data have been incorporated into Supplementary Figure 4 b in this resubmission.

4: The authors use dendritic cells from patients to analyse the effect of IL-37 variants. Are dendritic cells producing IL-37 and IL-37 target in periodontitis in vivo?
Response: Thanks to the reviewer for raising these interesting questions. Previous studies have reported that dendritic cells could produce IL-37 (Proc Natl Acad Sci U S A. 2014 Oct 21;111(42):15178-83. doi: 10.1073/pnas.1416714111. Epub 2014 Oct 7. Suppression of antigen-specific adaptive immunity by IL-37 via induction of tolerogenic dendritic cells. Luo Y, Cai X, Liu S, Wang S, Nold-Petry CA, Nold MF3, Bufler P, Norris D, Dinarello CA, Fujita M.). In addition, previous study also reported recombinant IL-37 could suppress DC maturation and cytokine production (IL-37b suppresses T cell priming by modulating dendritic cell maturation and cytokine production via dampening ERK/NF-κB/S6K signalings. Wu W, Wang W, Wang , Li W, Yu G, Li Z, Fang C, Shen Y, Sun Z, Han L, Yu J, Fang L, Chen S, Dong K, Han Z, Liu H, Luo Y, Feng X). This evidence demonstrated the feasibility and relevance for us to utilize dendritic cells for analyzing the effect of IL-37 variants. Specifically, to answer the question of the reviewer whether DC producing IL-37 in periodontitis, we performed the immunofluorescent staining of IL-37 and DC specific marker (CD11c) using human gingival tissue from periodontitis patient, our data indicated the CD11c positive cells also produce IL-37 which indicate that DC is one of the sources of IL-37 in human gingival tissues. We have incorporated the data into Supplementary Figure4c. Unfortunately, we could not answer the question if DC is the target of IL-37 target in periodontitis in vivo in this resubmission as our laboratory does not have necessary DC associated mice strains at this time point. But this is definitely an extremely interesting question to answer in the future.

5: The authors focused on IL-1β GCF levels to define genes associated with periodontitis severity, since it is considered a key prognostic factor in periodontitis. The contribution of other inflammatory cytokines involved in the pathogenesis (e.g. IL-17A) that could be influenced by the altered IL-37 function should be at least discussed. Figure 3A: the increased levels of the inflammatory mediators analyzed should be discussed.
Response: We really appreciate this reviewer's suggestion and have incorporated the influence of IL-37 to other cytokines besides IL-1β in our discussion. The impact of IL-37 variants on key regulatory cytokines, like IL-17 in periodontitis is currently under our investigation and may report in separate manuscript. We also discussed the cytokines data of Figure 3A in our discussion.

6: A more detailed analysis of the mouse models (Figure 6 and Figure 8) would be interesting. Is there a difference in IL-1β level in mice treated with IL-37, in the model shown in figure 6?
Response: We sincerely thank this reviewer's suggestion. We performed qPCR to quantitate the IL-1β mRNA level of the gingival tissues in our ligature induced periodontitis model with rhIL-37 treatment. We also assess the IL-1β protein level by IHC using anti-IL-1β antibody. Both data indicated that the IL-1β level in the gingival tissue in the ligature model is decreased after rhIL-37 treatment. We have incorporated these two data findings into Figure 6 f and g.

7: Importantly, the in vivo periodontitis model should be performed in V1IL-37 transgenic mice (Figure 8). Finally, is the immune infiltrate altered by IL-37 variants?
Response: We appreciate the reviewer for raising these constructive questions. This is exactly what we had anticipated using our transgenic mice (it takes UNC transgenic core facility and our group for almost two years to generate sufficient numbers of these WT and variants IL-37 transgenic mice for these experiments). However, just as you have suggested, we have performed *in vivo* ligature induced periodontitis model using WT and V1 IL-37 transgenic mice. The IL-1β level in gingival tissue has been quantitated by qPCR and showed a significant difference between WT IL-37 and V1 Tg mice, with higher levels of IL-1β in the V1 Tg mice. The bone alveolar bone loss has also been quantitated for both WT IL-37 and V1 Tg mice, demonstrating greater bone loss in the V1Tg mice. Both sets of data have been incorporated into Figure 8 g-i.

8: The text language and manuscript organization could be improved. There are typos.
Response: We appreciate the suggestion of this reviewer. We have revised to improve the writing of our manuscript.

For Reviewer #3 (Remarks to the Author):

1: In the abstract, it is not clear why different cut off values were selected to define high (quartiles) versus elevated IL-1B levels (percentiles / median).
Response: Thanks reviewer for this great suggestion. We have revised the manuscript to explain this clearly. However, due to the word limitation of abstract, we clarified this in the results instead of abstract. The reason for selecting quartiles is due to the data distribution that be seen in Figure 2 where the inflection of the tail of the distribution begins at Q4. The monotonic increase in IL-1b in the lower Q1 and Q2 were associated with the IL-1β locus. We also examined the upper 90th percentile which captures only the top of hyper-inflammatory distribution with similar results for the major *IL37* locus. When we examined the distribution between the 75th percentile and the 90th percentile, this increase in IL-1β was also associated with IL-37 rather than IL-1β SNPs. For this reason the Q4 seemed most appropriate for defining the IL-37- based hyper IL-1b response. Using GCF IL-1 β as a continuous variable also identified the IL-37 locus, but the distribution suggested that a categorical approach would be more appropriate.

2. Is IL37 expressed in cells from non-diseased periodontal tissue?
Response: This is a great question. According to the IHC staining using gingival tissue biopsies from health subjects, we observed the positive staining of IL-37 in epithelial and endothelial cells. Notably, non-diseased periodontal tissues do not have robust inflammatory cells such as plasma cell infiltration. Our hypothesis is that there are mainly constitutive IL-37 expression from epithelial and endothelial cells in healthy condition to maintain the inflammatory balance of periodontal tissue. Once chronic periodontitis occurs, robust plasma cells infiltrate and serve as another major source of IL-37 in periodontal tissues to suppress the inflammation. We are planning to test our hypothesis in separate project.

3. In the introduction, it would be helpful to add where the IL37 gene maps when it is first mentioned to make it clear that it is within the IL1 gene cluster on 2q13.
Response: Thanks for this great point, we have incorporated this information into abstract and the introduction.

4. In table 1, adding the sample size of the entire cohort under the entire sample title would help.
Response: Thank you, we have added those sample sizes to the header of Table 1.

5. Discovery GWAS section: the sentence beginning "The phenotypic variance..." does not make sense to me.
Response: This is a great point. We have revised the text to eliminate that statement.

6. Was any conditional analysis performed to confirm that there are independent signals across the IL-1 complex locus?
Response: This is an excellent suggestion. However, our stepwise analysis showed the IL37 as the dominant QTL with the other loci in the region contributing very little overall variance to the IL-1β response (Table 2). It would appear likely that the *IL1b* and perhaps the *IL1ra* locus, as well as an effector region located downstream of *IL1b* may interact with the *IL37* locus, but the magnitude would be relatively small. Therefore, we feel that a detailed analysis of haplotype context may be beyond the focus of this report.

7. I found Table 2 hard to interpret. I was expecting to see haploytpes but this seemed to be a diplotype analysis of genotype combinations. Haplotypes should be the alleles and would make it easier to interpret.
Response: Thank you for this suggestion, we have added in the allelic haplotypes to the figure to clarify.

8. The manuscript could be shortened by avoiding repetition and with some editing eg in sections 'Associatoin of IL37 variants with prevalent...,aggresive periodontitis,....ARIC populations'.
Response: We appreciate the reviewer's suggestion. We have revised our manuscript to avoid repetition.

9. I would like to see p-values in Table 6. I am concerned this data is being over-interpreted, given the wide confidence intervals for some groups. It detracts from the main message of the paper and I would advise moving it to supplementary material. I think it is also misleading to claim association in the discussion.
10. Have IL-1 genes been associated with CVD at genome-wide significance? Again, the section in the Discussion risks over-interpreting the findings
Response: Thank you for the suggestion. We agree that this is not the main focus of our manuscript and have moved it to the supplementary material (Supplementary Table 2). Although the IL-1 gene complex has been identified as being associated with various CVD outcomes in numerous candidate gene studies, it is not from agnostic GWAS analyses. However, our data are stronger than any of these previous reports and we would like to highlight that the inflammatory trait may extend to other conditions of which inflammation is a key determinant. We feel it is worth including because of the interest in the connection between IL-1β, and CVD, especially in light of the new CVD trials demonstrating efficacy with IL-1b inhibitors. But your point is well taken and we have moved this table to the supplement and minimized the discussion emphasizing the lack of genome-wide significance.

11. The Discussion is very long and sometimes a repetition of the results.
Response: Thanks reviewer for this suggestion. We have revised to shorten our discussion.

Reviewer #1 (Remarks to the Author):

All of my comments have been well addressed and the manuscript has become suitable for publication.

Reviewer #2 (Remarks to the Author):

The authors addressed my questions and comments in a satisfactory manner.

Reviewer #3 (Remarks to the Author):

None
